# *CELF2* is a candidate prognostic and immunotherapy biomarker in triple‐negative breast cancer and lung squamous cell carcinoma: A pan‐cancer analysis

**DOI:** 10.1111/jcmm.16791

**Published:** 2021-07-19

**Authors:** Libo Wang, Zaoqu Liu, Long Liu, Chunguang Guo, Dechao Jiao, Lifeng Li, Jie Zhao, Xinwei Han, Yuling Sun

**Affiliations:** ^1^ Department of Hepatobiliary and Pancreatic Surgery The First Affiliated Hospital of Zhengzhou University Zhengzhou China; ^2^ Institute of Hepatobiliary and Pancreatic Diseases Zhengzhou University Zhengzhou China; ^3^ Zhengzhou Basic and Clinical Key Laboratory of Hepatopancreatobiliary Diseases The First Affiliated Hospital of Zhengzhou University Zhengzhou China; ^4^ Department of Interventional Radiology The First Affiliated Hospital of Zhengzhou University Zhengzhou China; ^5^ Department of Endovascular Surgery The First Affiliated Hospital of Zhengzhou University Zhengzhou China; ^6^ Internet Medical and System Applications of National Engineering Laboratory Zhengzhou China; ^7^ Cancer Center The First Affiliated Hospital of Zhengzhou University Zhengzhou China; ^8^ Department of Pharmacy The First Affiliated Hospital of Zhengzhou University Zhengzhou China

**Keywords:** *CELF2*, immune infiltration, immunotherapy, lung squamous cell carcinoma, prognosis, triple‐negative breast cancer

## Abstract

CUGBP Elav‐like family member 2(*CELF2*) plays crucial roles in the development and activation of T cell. However, the impacts of *CELF2* on tumour‐infiltrating immune cells (TIICs) and clinical outcomes of tumours remain unclear. In this study, we found that elevated *CELF2* expression was markedly correlated with prolonged survival in multiple tumours, particularly in breast and lung cancers. Notably, *CELF2* only impacted the prognosis of triple‐negative breast cancer (TNBC) with lymph node metastasis. Further investigation showed *CELF2* expression was positively correlated with the infiltration abundance of dendritic cells (DCs), CD8+ T cells and neutrophils in breast invasive carcinoma (BRCA) and DCs in lung squamous cell carcinoma (LUSC). *CELF2* also had strong correlations with markers of diverse TIICs such as T cells, tumour‐associated macrophages and DCs in BRCA and LUSC. Importantly, *CELF2* was significantly associated with plenty of immune checkpoint molecules (ICMs) and outperformed five prevalent biomarkers including *PD*‐*1*, *PD*‐*L1*, *CTLA*‐*4*, *CD8* and tumour mutation burden in predicting immunotherapeutic responses. Immunohistochemistry also revealed lower protein levels of *CELF2* in TNBC and LUSC compared to normal tissues, and patients with high expression showed significantly prolonged prognosis. In conclusion, we demonstrated that increased *CELF2* expression was closely related to better prognosis and superior TIIC infiltration and ICM expression, particularly in BRCA and LUSC. *CELF2* also performed well in evaluating the immunotherapeutic efficacy, suggesting *CELF2* might be a promising biomarker.

## 1 INTRODUCTION

With the change in disease spectrum, tumours have become a major threat to people's health in recent years, placing a heavy burden on global public health. The latest statistical report shows that in 2021, there will be more than 1,898,160 new cancer cases and 608,570 cancer deaths in the United States, which has become the leading cause of death in developed countries. Among them, the incidence of lung cancer ranks second and has becoming the number one killer in tumour‐related disease for both men and women. In terms of women, breast cancer is the most common tumour type, with the second highest mortality rate for a long time, after lung cancer.^1^ Although the mortality rate of breast and lung cancer has decreased with the improvement of tumour diagnosis and treatment, the clinical outcomes remain unsatisfactory. For patients with lung cancer, the 5‐year survival rate is only 21%. Breast cancer exhibits a 5‐year survival rate of nearly 90%, while the 5‐year survival rate of distant metastatic breast cancer is only 28%.^1^ Hence, a better understanding the specific mechanisms of breast and lung cancer oncogenesis and progression, as well as to find more accurate novel biomarkers that can be used for clinical and therapeutic management, is urgently needed.

Tumour microenvironment (TME) is the cellular environment for tumour cell growth, in which tumour‐infiltrating immune cells (TIICs), an important component, play a dominant role.^2^ For example, tumour‐associated macrophages (TAMs) can exacerbate tumour progression by promoting tumour angiogenesis, metastasis and immune escape.^3^ Dendritic cells (DCs) conduce to tumour metastasis by reducing CD8+ T‐cell cytotoxicity and enhancing regulatory T (Treg) cell responses.^4^, ^5^ The past decade has witnessed encouraging advances in immunotherapy represented by immune checkpoint inhibitors (ICIs) has revolutionized the therapeutic paradigm of most tumours, especially non‐small‐cell lung cancer, triple‐negative breast cancer (TNBC), advanced melanoma and bladder cancer.^6^, ^7^, ^8^, ^9^ While ICIs target the interactions between immune and tumour cells within the TME, certain alterations that occur in the TME can also affect the responsiveness to immunotherapy.^3^ Recent studies have confirmed that key biological processes such as autophagy, hypoxia and ferroptosis, as well as some molecular alterations, can contribute to the immunotherapeutic efficacy and prognosis of cancer patients by affecting the distributions and interactions of distinct immune cell subsets in the TME.^10^, ^11^, ^12^, ^13^ To date, there are still few cancer patients who can benefit from immunotherapy, and thus, it is essential to explore additional therapeutic targets.

CELF (CUGBP Elav‐like family) proteins are RNA binding protein of shuttle nucleoplasm characterized by three RNA recognition motifs. In humans, CELF contains six known isoforms, *CELF1* to *CELF6*, which are further divided into two subgroups according to their amino acid sequencing similarity. One group consists of *CELF3*‐*6* and is largely restricted to neurons and a few other tissues. The other group includes *CELF1* and *CELF2*, which are commonly expressed in most tissues, but are expressed differently during development and differentiation.^14^ Previous studies have demonstrated that *CELF2* could regulate multiple steps of RNA processing, such as pre‐mRNA splicing, RNA editing, polyadenylation, mRNA stability and translation.^14^, ^15^ Over the past decade, substantial studies have confirmed that *CELF2* played a tumour suppressor role in breast cancer, lung cancer, hepatocellular carcinoma, gastric cancer, ovarian cancer, glioma and acute myeloid leukaemia, suggesting that it can be used as a candidate biomarker to predict cancer prognosis.^16^, ^17^, ^18^, ^19^, ^20^, ^21^, ^22^, ^23^ Additionally, *CELF2* expression was significantly elevated in developing thymocytes and activated T cells and promoted T‐cell receptor expression and signalling through alternative splicing.^24^, ^25^, ^26^ However, the comprehensive understanding of the impacts of *CELF2* on the tumour immune microenvironment remains unknown.

Herein, we delineated the expression and prognostic landscape of *CELF2* across human cancers. The relationships between *CELF2* and TIICs as well as immune checkpoint molecules (ICMs) were further explored. In addition, we also recruited two immunotherapeutic cohorts and evaluated the power of *CELF2* for predicting the responses to immunotherapy. Overall, our study provides a reference and direction for understanding the crucial role of *CELF2* in the immune microenvironment of pan‐cancer, as well as reveals the potential mechanism thereby *CELF2* affects anti‐tumour immunity and cancer immunotherapy.

## 2 METHODS

### 2.1 *CELF2* expression across human cancers in Oncomine

Oncomine (https://www.oncomine.org/resource/login.html) is a web‐based data mining platform that assembles 86,733 samples and 715 gene expression data sets together.^27^ The mRNA expression levels of *CELF2* in various cancer types were detected using Oncomine database with the following conditions: *p*‐value: 0.001, fold change: 1.5, and gene rank: all.

### 2.2 Survival analysis in PrognoScan and Kaplan‐Meier plotter

The biological correlations between *CELF2* expression and patient survival in pan‐cancer were evaluated via PrognoScan (http://dna00.bio.kyutech.ac.jp/PrognoScan/index.html) and Kaplan‐Meier plotter (http://kmplot.com/analysis/)^28^, ^29^. The PrognoScan database is publicly available to assess the prognostic value of genes by meta‐analysing a large collection of published cancer microarray data. The Kaplan‐Meier plotter, which includes data from the Gene Expression Omnibus (GEO), The Cancer Genome Atlas (TCGA) and European Genome‐phenome Atlas (EGA), provides an easy way to explore the impact of 54,000 genes on survival in 21 human cancers, with a large cohort of breast (*n* = 7830), ovarian (*n* = 2190), lung (*n* = 3452) and gastric (*n* = 1440) cancers. We therefore evaluated the correlations between *CELF2* expression and patient survival in breast, ovarian, lung and gastric cancers and further analysed the impacts of *CELF2* expression on the outcomes of breast cancer patients with different clinicopathological characteristics. The hazard ratios (HR) with 95% confidence intervals (95% CI) and log‐rank *p*‐value (< 0.05 is considered to be significant) was also calculated.

### 2.3 Correlations between *CELF2* expression and immune cell infiltration in TIMER

TIMER (https://cistrome.shinyapps.io/timer/) is a comprehensive resource that applies a deconvolution method to infer the abundance of TIICs from the TCGA database.^30^ We analysed *CELF2* expression level in different cancer types by the DiffExp module of TIMER. Afterwards, we explored the Spearman correlations between *CELF2* expression and tumour purity as well as the abundance of six TIICs including B cells, CD4+ T cells, CD8+ T cells, neutrophils, macrophages and DCs in 32 cancers using the gene module of TIMER.

In addition, we also explored the correlations between *CELF2* and several immune cell markers. The gene markers of TIICs including CD8+ T cells, T cells (general), B cells, monocytes, TAMs, M1 macrophages, M2 macrophages, neutrophils, natural killer (NK) cells, DCs, T‐helper 1 (Th1) cells, T‐helper 2 (Th2) cells, follicular helper T (Tfh) cells, T‐helper 17 (Th17) cells, Treg and exhausted T cells were referenced from previous studies.^31^, ^32^, ^33^ In this part, we focused on analysing breast invasive carcinoma (BRCA) and lung squamous cell carcinoma (LUSC), with lung adenocarcinoma (LUAD) as a control. Finally, we further explored the correlations between *CELF2* and ICMs in various cancer types using the Gene_Corr module of the TIMER2.0 website.^34^ The ICMs were derived from previous studies.^35^, ^36^ Notably, the Spearman correlation coefficients presented in the heatmap were adjusted for tumour purity.

### 2.4 Gene correlation analysis in GEPIA

The online database GEPIA (http://gepia.cancer‐pku.cn/index.html) is an interactive analysis tool that contains RNA‐seq data from 9736 tumour and 8587 normal samples from the TCGA and Genotype‐Tissue Expression (GTEx) data set.^37^ We used GEPIA to explore the relationships between *CELF2* and TIIC‐related markers in BRCA, LUSC and LUAD. The Spearman method was applied to determine the correlation coefficient.

### 2.5 Evaluation of immunotherapeutic biomarkers

We finally recruited two immunotherapeutic cohorts: (1) a cohort of 38 metastatic melanoma patients treated with anti‐PD‐1 monoclonal antibody (GSE78220 cohort)^12^, ^38^ and (2) a cohort of 144 melanoma patients treated with anti‐PD‐1 monoclonal antibody (Van Allen cohort).^39^ In addition, to explore the power of *CELF2* as an immunotherapeutic biomarker, we evaluated the performance of *CELF2* in predicting immunotherapy response in the two cohorts and further compared with five other well‐studied biomarkers, including *PD*‐*1*, *PD*‐*L1*, *CTLA*‐*4*, *CD8* and tumour mutation burden (TMB).^40^, ^41^, ^42^ We used the receiver operator characteristic (ROC) curves and the area under the ROC curve (AUC) to measure the predictive accuracy of different biomarkers for predicting the responses to immunotherapy.

### 2.6 Tissue microarray and immunohistochemistry staining

Human tissue microarrays of TNBC (BRC1601; Shanghai Superbiotek Pharmaceutical Technology, Shanghai, China) and LUSC (HLugS180Su02; Shanghai Outdo Biotechnology, Shanghai, China) were purchased. The clinical characteristics of 80 paired TNBC and 90 paired LUSC specimens were downloaded from the company websites. Immunohistochemistry (IHC) was performed using anti‐*CELF2* (ab186430, 1:500) antibody. Staining percentage scores were classified as follows: 1 (1%–25%), 2 (26%–50%), 3 (51%–75%) and 4 (76%–100%), and staining intensity was scored 0 (signalless colour) to 3 (light yellow, brown and dark brown). The stained tissues were scored by three individuals blinded to the clinical parameters, and the IHC scores were determined by percentage and intensity scores.

### 2.7 Statistical analysis

Differential expression of *CELF2* in TIMER was explored using the Wilcoxon rank‐sum test. The results produced by Oncomine were displayed with P‐value, fold change and gene rank. Survival was assessed using PrognoScan and Kaplan‐Meier plotter. The correlations between two continuous variables were evaluated by Spearman's correlation and statistical significance in TIMER, TIMER2.0 and GEPIA. The strength of the correlation was determined using the following guidelines for absolute values: 0.00–0.19, very weak; 0.20–0.39, weak; 0.40–0.59, moderate; 0.60–0.79, strong; and 0.80–1.00, very strong. Data processing, statistical analysis and plotting of the immunotherapy and tissue microarray cohorts were conducted in R 4.0.2 software. Kaplan‐Meier survival analysis was performed by survival R package, and the optimal cut‐off value was determined by survminer R package. The ROC curves were plotted by pROC R package. *p* < 0.05 was considered statistically significant.

## 3 RESULTS

### 3.1 The mRNA expression levels of *CELF2* in pan‐cancer

**FIGURE 1:**
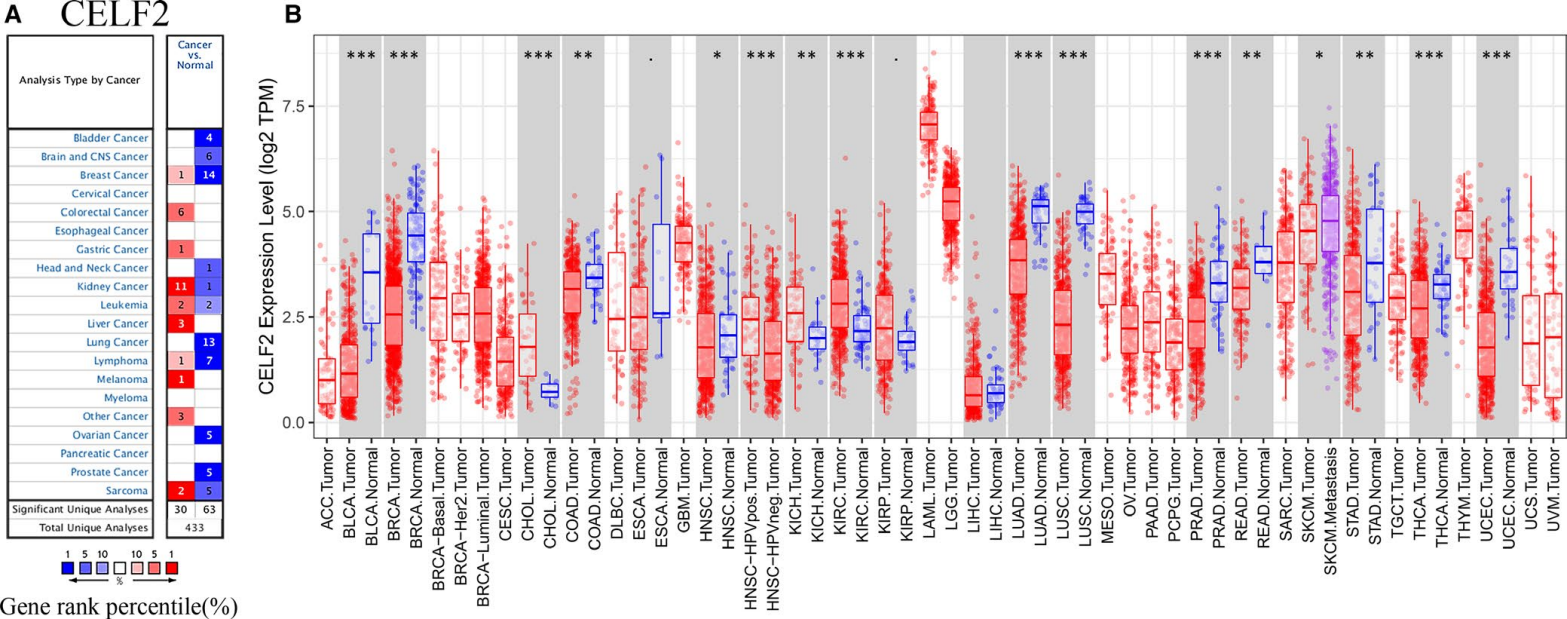
*CELF2* expression levels in different types of human cancers. (A) Increased or decreased *CELF2* in data sets of different cancers compared with normal tissues in Oncomine database. (B) Human *CELF2* expression levels in different tumour types from the TCGA database were determined by TIMER (*p < 0.05, **p < 0.01, ***p < 0.001)

We first analysed the expression levels of *CELF2* mRNA in pan‐cancer using Oncomine database. The results demonstrated that *CELF2* was significantly elevated in colorectal, gastric, kidney, leukaemia, liver and melanoma cancers relative to their matched normal tissues. In contrast, we also found that *CELF2* was lower in bladder, brain and central nervous system, breast, head and neck, lung, lymphoma, ovarian, prostate and sarcoma cancers compared with normal tissues (Figure 1A). Detailed expression results of *CELF2* in specific tumours are shown in Supplementary Table 1.

To further assess *CELF2* expression in different cancer types, we used the TIMER tool to analyse RNA‐seq data from the TCGA database. We found that *CELF2* expression was significantly down‐regulated in bladder urothelial carcinoma (BLCA), BRCA, colon adenocarcinoma (COAD), head and neck squamous cell carcinoma (HNSC), LUAD, LUSC, prostate adenocarcinoma (PRAD), rectum adenocarcinoma (READ), stomach adenocarcinoma (STAD), thyroid carcinoma (THCA) and uterine corpus endometrial carcinoma (UCEC) relative to their respective adjacent normal tissues. In contrast, significantly higher expression of *CELF2* was only found in cholangiocarcinoma (CHOL), kidney chromophobe (KICH) and kidney renal clear cell carcinoma (KIRC). The differential *CELF2* expression in tumours and adjacent normal tissue samples in the TCGA database is shown in Figure 1B.

### 3.2 Potential prognostic value of *CELF2* in pan‐cancer

**FIGURE 2:**
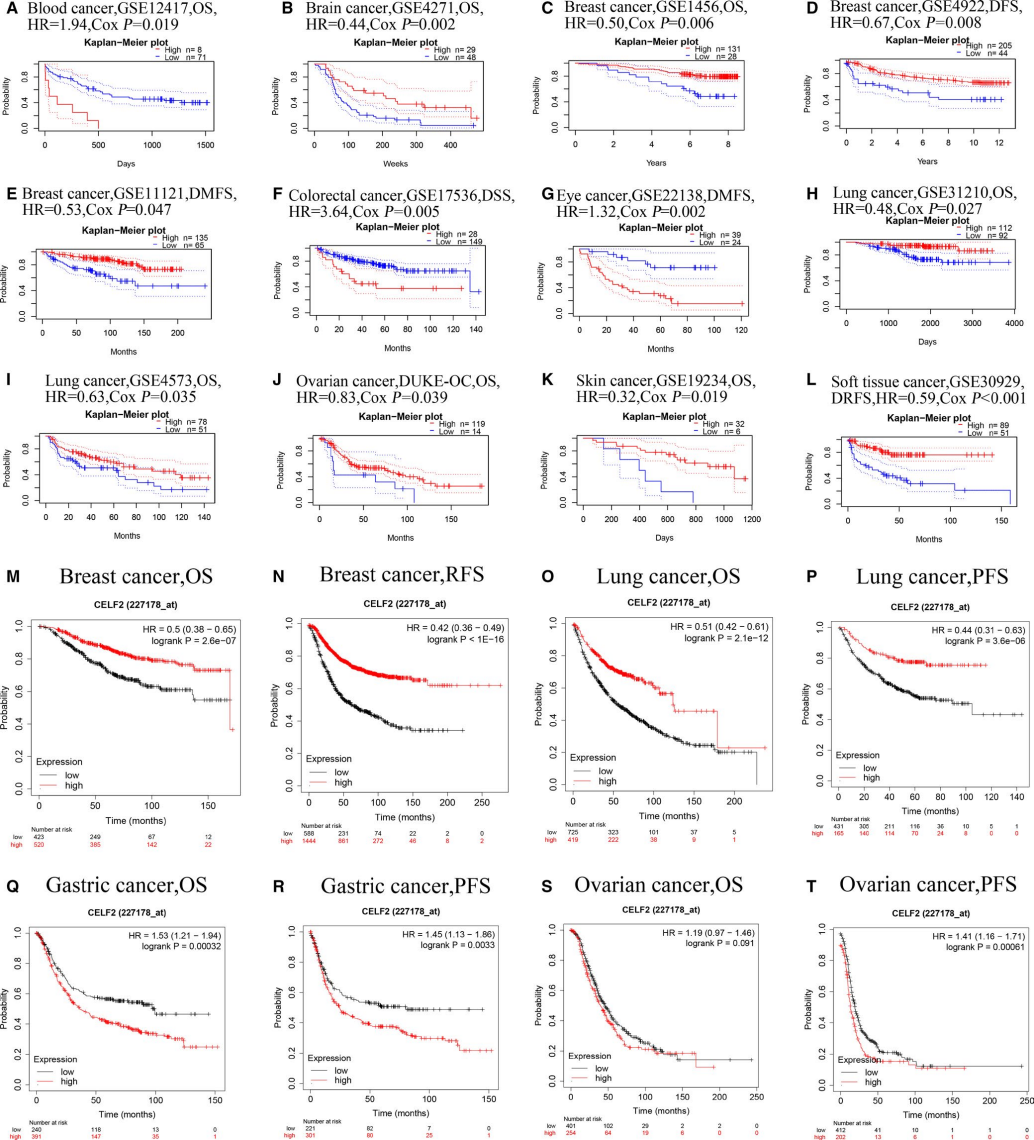
Representative Kaplan‐Meier survival curves comparing the high and low expression of *CELF2* in multiple types of cancer in PrognoScan (A–L) and Kaplan‐Meier plotter database (M–T). (A, B) Survival curves of OS in blood cancer cohort (GSE12417, *n* = 79) and brain cancer cohort (GSE4271, *n* = 77). (C–E) Survival curves of OS (GSE1456, *n* = 159), DFS (GSE4922, *n* = 249) and DMFS (GSE11121, *n* = 200) in three breast cancer cohorts. (F) Survival curve of DSS in colorectal cancer cohort (GSE17536, *n* = 177). (G) Survival curve of DMFS in eye cancer cohort (GSE22138, *n* = 63). (H–I) Survival curves of OS in two lung cancer cohorts (GSE31210, *n* = 204; GSE4573, *n* = 129). (J–K) Survival curves of OS in ovarian cancer cohort (DUKE‐OC, *n* = 133) and skin cohort (GSE19234, *n* = 38). (L) Survival curve of DRFS in soft tissue cancer cohort (GSE30929, *n* = 140). (M, N) OS and RFS survival curves of breast cancer (*n* = 1879; *n* = 4929). (O, P) OS and PFS survival curves of lung cancer (*n* = 1925; *n* = 982). (Q, R) OS and PFS survival curves of gastric cancer (*n* = 875; *n* = 640). (S, T) OS and PFS survival curves of ovarian cancer (*n* = 1656; *n* = 1435). OS, overall survival; DFS, disease‐free survival; DMFS, distant metastasis‐free survival; DSS, disease‐specific survival; DRFS, distant relapse‐free survival; RFS, relapse‐free survival; PFS, progression‐free survival

We next investigated the impact of *CELF2* expression on the prognosis of different cancers using PrognoScan (Supplementary Table 2). The results revealed a significant correlation between *CELF2* expression and the survival of patients with a variety of tumours, including blood, brain, breast, colorectal, eye, lung, ovarian, skin and soft tissue cancers. Representative survival curves for each tumour are shown in Figure 2A–L. Notably, there were 24 and 15 cohorts, respectively, that showed high expression of *CELF2* as a protective factor in breast and lung cancer (Figure 2C–E, H–I, Supplementary Table 2). These results suggested that the expression of *CELF2* had a non‐negligible impact on the prognosis of breast and lung cancers.

After PrognoScan, we also used Kaplan‐Meier plotter database to evaluate the prognostic value of *CELF2*. The better prognosis in breast cancer (overall survival (OS): HR = 0.5, 95% CI = 0.38 to 0.65, log‐rank *p* = 2.6e‐7; relapse‐free survival (RFS): HR = 0.42, 95% CI = 0.36 to 0.49, log‐rank *p* < 1e‐16) and lung cancer (OS: HR = 0.51, 95% CI = 0.42 to 0.61, log‐rank *p* = 2.1e‐12; progression‐free survival (PFS): HR = 0.44, 95% CI = 0.31 to 0.63, log‐rank *p* = 3.6e‐6) was shown in patients with higher *CELF2* expression (Figure 2M–P). In contrast, we found that increased *CELF2* expression correlated with a dismal prognosis in gastric cancer (OS: HR = 1.53, 95% CI = 1.21 to 1.94, log‐rank *p* = 0.00032; PFS: HR = 1.45, 95% CI = 1.13 to 1.86, log‐rank *p* = 0.0033) and ovarian cancer (PFS: HR = 1.41, 95% CI = 1.16 to 1.71, log‐rank *p* = 0.00061) (Figure 2Q, R, T). However, despite the OS of ovarian cancer also presented this trend, it was not statistically significant (OS: HR = 1.19, 95% CI = 0.97 to 1.46, log‐rank *p* = 0.091; Figure 2S). Taken together, these results in PrognoScan and Kaplan‐Meier plotter simultaneously illustrated that *CELF2* was related to its better survival in breast and lung cancers.

### 3.3 Elevated *CELF2* expression impacted the prognosis of triple‐negative breast cancer patients with lymphatic metastasis

TNBC is a special type of breast cancer in which estrogen receptor (ER), progesterone receptor (PR) and human epidermal growth factor receptor 2 (HER2) are negative, which is characteristic by the lack of effective therapeutic targets, and a high degree of malignant, easy to metastasis and relapse.^43^ As we found *CELF2* was significantly down‐regulated in breast cancer, and its expression was closely related to better prognosis. To better understand the specific mechanism by which *CELF2* affects prognosis in breast cancer, by integrating clinicopathological information from the Kaplan‐Meier plotter database, we explored the prognostic value of *CELF2* in stratified populations. We found that *CELF2* expression exerted a positive effect on both OS and RFS in breast cancer and was significantly correlated with ER, PR, HER2 status, subtype, grade, lymph node status and TP53 status of patients (Table 1). Specifically, high *CELF2* expression was dramatically associated with prolonged OS and RFS in breast cancer patients with ER negative, PR negative, HER2 negative, lymph node positive and grade 3 (OS and RFS: HR <1 and *p* < 0.05). Meanwhile, we also found no significant correlations between *CELF2* expression and OS or RFS in patients with ER positive (OS: HR = 0.52, *p* = 0.06), PR positive (RFS: HR = 0.67, *p* = 0.0744), lymph node negative (OS: HR = 0.68, *p* = 0.3503; RFS: HR = 0.65, *p* = 0.0261), grade 1 (OS: HR = 0.29, *p* = 0.2841; RFS: HR = 1.72, *p* = 0.3512) and grade 2 (OS: HR = 2.38, *p* = 0.1271). Considering the absence of OS in PR‐positive patients, we further evaluated the influence of *CELF2* on distant metastasis‐free survival (DMFS) of PR‐positive patients and revealed that *CELF2* expression was not significantly associated with DMFS (DMFS: HR = 0.62, *p* = 0.3571). Our results indicated that *CELF2* played a protective role in TNBC patients with lymph node metastasis and higher grade, suggesting that *CELF2* might affect the prognosis of TNBC patients through lymph node metastasis in these individuals.

**TABLE 1:** Correlation of *CELF2* mRNA expression and clinical prognosis in breast cancer with different clinicopathological factors by Kaplan‐Meier plotter

Clinicopathological characteristics	Overall survival (*n* = 1879)			Relapse‐free survival (*n* = 4929)		
	*N*	Hazard ratio	*p* value	*N*	Hazard ratio	*p* value
ER status‐IHC						
ER positive	754	0.52 (0.26–1.04)	0.06	2633	0.66 (0.49–0.87)	**0.0033**
ER negative	520	0.48 (0.31–0.75)	**0.001**	1190	0.58 (0.43–0.8)	**0.00063**
ER status‐array						
ER positive	1309	0.5 (0.34–0.74)	**0.00038**	3768	0.42 (0.35–0.51)	**<1e‐16**
ER negative	570	0.5 (0.33–0.74)	**0.00047**	1161	0.42 (0.33–0.54)	**3.1e‐12**
PR status‐IHC						
PR positive	156	—	—	926	0.67 (0.43–1.04)	0.074
PR negative	291	0.43 (0.22–0.85)	**0.012**	925	0.54 (0.38–0.76)	**0.00034**
HER2 status‐array						
HER2 positive	420	0.46 (0.28–0.76)	**0.0021**	882	0.43 (0.32–0.59)	**2.6e‐08**
HER2 negative	1459	0.51 (0.37–0.7)	**3.0e−05**	4047	0.41 (0.34–0.49)	**<1e‐16**
Intrinsic subtype						
Basal	404	0.51 (0.32–0.81)	**0.0035**	846	0.37 (0.27–0.5)	**4.0e‐11**
Luminal A	794	0.52 (0.32–0.87)	**0.010**	2277	0.44 (0.35–0.56)	**1.1e‐11**
Luminal B	515	0.41 (0.22–0.78)	**0.0048**	1491	0.38 (0.28–0.51)	**1.3e‐10**
HER2+	166	0.44 (0.22–0.9)	**0.022**	315	0.41 (0.23–0.75)	**0.0027**
Lymph node status						
Lymph node positive	452	0.5 (0.31–0.82)	**0.0047**	1656	0.56 (0.44–0.71)	**1.6e‐06**
Lymph node negative	726	0.68 (0.3–1.54)	0.35	2368	0.65 (0.44–0.95)	**0.026**
Grade						
1	175	0.29 (0.03–3.23)	0.28	397	1.72 (0.54–5.5)	0.35
2	443	2.38 (0.75–7.5)	0.13	1177	0.53 (0.32–0.9)	**0.016**
3	586	0.53 (0.31–0.91)	**0.018**	1300	0.5 (0.36–0.68)	**8.6e‐06**
TP53 status						
Mutated	130	3.04 (0.63–14.62)	0.15	188	0.4 (0.2–0.79)	**0.0063**
Wild type	197	—	—	273	2.25 (0.95–5.3)	0.057
Pietenpol subtype						
Basal‐like1	103	0.36 (0.14–0.98)	**0.036**	251	0.37 (0.21–0.66)	**0.00053**
Basal‐like2	58	2.25 (0.49–10.28)	0.28	101	0.48 (0.23–1.01)	**0.047**
Immunomodulatory	149	4.48 (0.97–20.74)	**0.036**	300	0.54 (0.23–1.25)	0.14
Mesenchymal	114	0.32 (0.12–0.84	**0.015**	211	0.38 (0.21–0.66)	**0.00043**
Mesenchymal stem‐like	39	0.31 (0.1–0.98)	**0.035**	81	0.38 (0.15–0.98)	**0.038**
Luminal androgen receptor	116	0.28 (0.11–0.72)	**0.0052**	253	0.31 (0.18–0.53)	**5.9e‐06**

Bold values indicate *p* < 0.05.

### 3.4 *CELF2* expression correlated with the immune infiltration in breast cancer and lung squamous cell carcinoma

**FIGURE 3:**
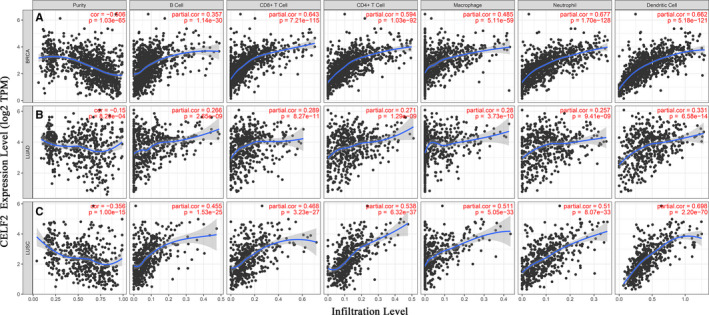
Correlations of *CELF2* expression with immune infiltration levels in BRCA (breast invasive carcinoma), LUAD (lung adenocarcinoma) and LUSC (lung squamous cell carcinoma). (A) *CELF2* expression is significantly negatively related to tumour purity and has moderate to strong positive correlations with infiltrating levels of CD8+ T cells, CD4+ T cells, macrophages, neutrophils and dendritic cells in BRCA, other than B cells. (B) *CELF2* expression has very weak correlation with tumour purity and weak correlations with infiltrating levels of B cells, CD8+ T cells, CD4+ T cells, macrophages, neutrophils and dendritic cells in LUAD. (C) *CELF2* expression is significantly negatively related to tumour purity and has moderate to strong positive correlations with infiltrating levels of B cells, CD8+ T cells, CD4+ T cells, macrophages, neutrophils and dendritic cells in LUSC

Multiple studies have confirmed that *CELF2* exerts tumour suppressive effects in most tumours, which is significantly increased in developing thymocytes and activated T cells, but its comprehensive understanding within the TME remains unknown.^16^, ^17^, ^18^, ^19^, ^20^, ^21^, ^22^, ^23^, ^24^, ^25^, ^26^ Therefore, it is necessary to investigate the relationships between *CELF2* expression and TIIC infiltration in the TME. In this study, we assessed the correlations of *CELF2* expression with the abundance of TIICs in 39 tumour types from the TIMER database. A significant correlation was shown between *CELF2* expression and tumour purity in 28 tumours (Figure 3 and Supplementary Figure 1). In addition, *CELF2* expression was also significantly associated with infiltration levels of B cells in 25 cancers, CD4+ T cells in 29 cancers, CD8+ T cells in 33 cancers, macrophages and DCs in 29 cancers and neutrophils in 32 cancers (Figure 3 and Supplementary Figure 1).

Given that *CELF2* expression was significantly correlated with diverse levels of immune infiltration in most of cancer types and combined with the results of *CELF2* expression and prognostic analysis in pan‐cancer, we selected subjects by setting the following criteria: (1) *CELF2* was significantly up‐ or down‐regulated in Oncomine and TIMER database at the same time; (2) *CELF2* had a consistent prognostic value in PrognoScan and Kaplan‐Meier plotter database; (3) *CELF2* expression was negatively associated with tumour purity and correlated with more than two TIIC levels. Interestingly, we found that *CELF2* expression was significantly related to a favourable prognosis and a high infiltration abundance of TIICs in breast and lung cancers. In BRCA, *CELF2* expression had a significant moderate to strong positive correlation with the infiltration levels of B cells (*r* = 0.357, *p* = 1.14E‐30), CD8+ T cells (*r* = 0.643, *p* = 7.21E‐115), CD4+ T cells (*r* = 0.594, *p* = 1.03E‐92), macrophages (*r* = 0.485, *p* = 5.11E‐59), neutrophils (*r* = 0.677, *p* = 1.70E‐128) and DCs (*r* = 0.662, *p* = 5.18E‐121) (Figure 3A). Similarly, there were obviously positive correlations with the infiltration levels of B cells (*r* = 0.455, *p* = 1.53E‐25), CD8+ T cells (*r* = 0.468, *p* = 3.23E‐27), CD4+ T cells (*r* = 0.538, *p* = 6.32E‐37), macrophages (*r* = 0.511, *p* = 5.05E‐33), neutrophils (*r* = 0.51, *p* = 8.07E‐33) and DCs (*r* = 0.698, *p* = 2.20E‐70) in LUSC (Figure 3C). However, in LUAD, although the correlations between *CELF2* with TIICs were also statistically significant, their correlations were very weak (Figure 3B). Of particular note where the correlation coefficients were greater than 0.6 between *CELF2* with CD8+ T cells, DCs and neutrophils in BRCA, as well as DCs in LUSC, indicating *CELF2* plays a crucial role in immune infiltration of these cells.

### 3.5 Expression correlations between *CELF2* and immune marker sets

**FIGURE 4:**
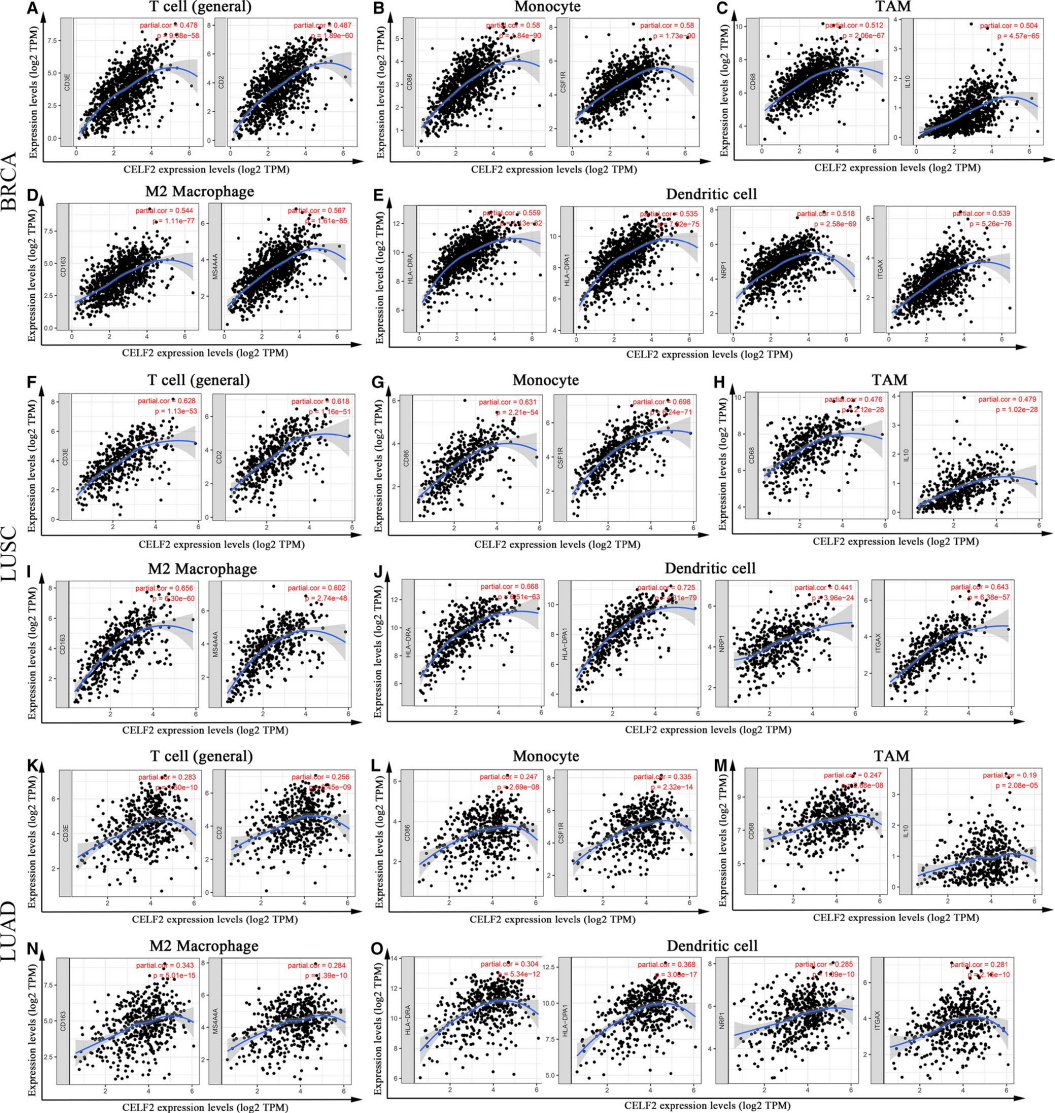
*CELF2* expression correlated with immune marker sets in BRCA (breast invasive carcinoma), LUSC (lung squamous cell carcinoma) and LUAD (lung adenocarcinoma). Markers include *CD3E* and *CD2* of T cell (general); *CD86* and *CSF1R* of monocyte; *CD68* and *IL10* of TAM (tumour‐associated macrophage); *CD163* and *MS4A4A* of M2 macrophage; *HLA*‐*DRA*, *HLA*‐*DPA1*, *NRP1* and *ITGAX* of dendritic cell. (A–E) Scatterplots of correlations between *CELF2* expression and gene markers of T cell (general) (A), monocyte (B), TAM (C), M2 macrophages (D) and dendritic cell (E) in BRCA. (F–J) Scatterplots of correlations between *CELF2* expression and gene markers of T cell (general) (F), monocyte (G), TAM (H), M2 macrophages (I) and dendritic cell (J) in LUSC. (K–O) The LUAD as the control group showed that *CELF2* expression has weak correlation with immune marker sets of T cell (general) (K), monocyte (L), TAM (M), M2 macrophages (N) and dendritic cell (O) in LUAD

In view of the relationships between *CELF2* expression and multiple TIIC infiltration levels in BRCA and LUSC, we further validated the correlations based on the marker gene sets of TIICs. We evaluated the correlations of *CELF2* expression with marker levels in specific immune cell subsets, including CD8+ T cells, T cells (general), B cells, monocytes, TAMs, M1 and M2 macrophages, neutrophils, NK cells and DCs in BRCA and LUSC, using LUAD as a control (Table 2 and Figure 4). Considering the prominent role played by *CELF2* during T‐cell development, we also focused on the correlations of *CELF2* with different functional T cells such as Th1, Th2, Tfh, Th17, Treg and exhausted T cells. After adjusting for tumour purity, we found that *CELF2* expression was still related to most of these marker sets, such as T‐cell markers (*CD3E* and *CD2*), monocyte markers (*CD86* and *CSF1R*), TAM markers (*CD68* and *IL10*), M2 macrophages markers (*CD163* and *MS4A4A*) and DC markers (*HLA*‐*DRA*, *HLA*‐*DPA1*, *NRP1* and *ITGAX*) in BRCA and LUSC (*p* < 0.0001 and *r* > 0.6; Figure 4A–J). In contrast, the majority of correlations between *CELF2* and individual immune cell markers in LUAD were not statistically significant, and the rare statistically significant coefficients were also less than 0.3, suggesting a relatively weak correlations (Table 2 and Figure 4K–O).

**TABLE 2:** Correlation analysis between *CELF2* and relate genes and markers of immune cells in TIMER

Description	Gene markers	BRCA				LUSC				LUAD			
		None		Purity		None		Purity		None		Purity	
		Cor	*p*	Cor	*p*	Cor	*p*	Cor	*p*	Cor	*p*	Cor	*p*
CD8+ T cell	CD8A	0.605	***	0.477	***	0.595	***	0.541	***	0.21	***	0.163	**
	CD8B	0.528	***	0.392	***	0.505	***	0.483	***	0.095	0.031	0.05	0.266
T cell (general)	CD3D	0.573	***	0.417	***	0.605	***	0.529	***	0.167	**	0.1	0.027
	CD3E	0.62	***	0.478	***	0.687	***	0.628	***	0.318	***	0.283	***
	CD2	0.621	***	0.487	***	0.677	***	0.618	***	0.295	***	0.256	***
B cell	CD19	0.447	***	0.277	***	0.521	***	0.42	***	0.196	***	0.152	**
	CD79A	0.487	***	0.312	***	0.525	***	0.422	***	0.193	***	0.151	**
Monocyte	CD86	0.651	***	0.58	***	0.684	***	0.631	***	0.295	***	0.247	***
	CD115 (CSF1R)	0.668	***	0.58	***	0.741	***	0.698	***	0.372	***	0.335	***
TAM	CCL2	0.496	***	0.383	***	0.506	***	0.45	***	0.138	*	0.09	0.045
	CD68	0.583	***	0.512	***	0.545	***	0.476	***	0.287	***	0.247	***
	IL10	0.587	***	0.504	***	0.535	***	0.479	***	0.249	***	0.19	***
M1 macrophage	INOS (NOS2)	0.234	***	0.243	***	0.15	**	0.178	***	0.154	**	0.139	*
	IRF5	0.318	***	0.25	***	0.149	**	0.126	*	0.155	**	0.124	*
	COX2 (PTGS2)	0.491	***	0.392	***	0.086	0.056	0.024	0.6	0.066	0.135	0.073	0.105
M2 macrophage	CD163	0.598	***	0.544	***	0.703	***	0.656	***	0.378	***	0.343	***
	VSIG4	0.52	***	0.442	***	0.632	***	0.577	***	0.261	***	0.22	***
	MS4A4A	0.649	***	0.567	***	0.658	***	0.602	***	0.33	***	0.284	***
Neutrophils	CD66b (CEACAM8)	0.02	0.502	0.045	0.157	0.167	**	0.163	**	0.287	***	0.288	***
	CD11b (ITGAM)	0.535	***	0.453	***	0.715	***	0.674	***	0.344	***	0.312	***
	CCR7	0.55	***	0.391	***	0.672	***	0.614	***	0.375	***	0.346	***
Natural killer cell	KIR2DL1	0.299	***	0.204	***	0.234	***	0.191	***	0.051	0.25	0.033	0.461
	KIR2DL3	0.303	***	0.207	***	0.312	***	0.269	***	0.073	0.099	0.03	0.511
	KIR2DL4	0.338	***	0.244	***	0.252	***	0.188	***	−0.057	0.198	−0.102	0.024
	KIR3DL1	0.38	***	0.28	***	0.421	***	0.376	***	0.12	*	0.09	0.045
	KIR3DL2	0.408	***	0.307	***	0.352	***	0.3	***	0.098	0.027	0.041	0.358
	KIR3DL3	0.201	***	0.143	***	0.106	0.017	0.093	0.042	−0.012	0.785	−0.034	0.451
	KIR2DS4	0.285	***	0.197	***	0.291	***	0.264	***	0.117	*	0.085	0.058
Dendritic cell	HLA‐DPB1	0.572	***	0.413	***	0.781	***	0.741	***	0.394	***	0.364	***
	HLA‐DQB1	0.461	***	0.327	***	0.575	***	0.51	***	0.324	***	0.291	***
	HLA‐DRA	0.662	***	0.559	***	0.718	***	0.668	***	0.343	***	0.304	***
	HLA‐DPA1	0.646	***	0.535	***	0.765	***	0.725	***	0.396	***	0.368	***
	BDCA‐1 (CD1C)	0.573	***	0.428	***	0.52	***	0.42	***	0.382	***	0.351	***
	BDCA‐4 (NRP1)	0.596	***	0.518	***	0.512	***	0.441	***	0.298	***	0.285	***
	CD11c (ITGAX)	0.622	***	0.539	***	0.695	***	0.643	***	0.319	***	0.281	***
Th1	T‐bet (TBX21)	0.584	***	0.443	***	0.687	***	0.635	***	0.278	***	0.241	***
	STAT4	0.671	***	0.549	***	0.654	***	0.596	***	0.281	***	0.25	***
	STAT1	0.445	***	0.417	***	0.392	***	0.347	***	0.16	**	0.129	*
	IFN‐γ (IFNG)	0.47	***	0.357	***	0.375	***	0.328	***	0.056	0.204	0.006	0.896
	TNF‐α (TNF)	0.313	***	0.271	***	0.293	***	0.201	***	0.136	*	0.077	0.086
Th2	GATA3	−0.246	***	−0.142	***	0.391	***	0.327	***	0.227	***	0.185	***
	STAT6	0.223	***	0.195	***	0.226	***	0.241	***	0.394	***	0.425	***
	STAT5A	0.415	***	0.311	***	0.668	***	0.62	***	0.39	***	0.363	***
	IL13	0.24	***	0.185	***	0.398	***	0.367	***	0.063	0.152	0.029	0.516
Tfh	BCL6	0.279	***	0.277	***	0.083	0.064	0.134	*	0.274	***	0.276	***
	IL21	0.372	***	0.29	***	0.39	***	0.344	***	0.105	0.017	0.082	0.068
Th17	STAT3	0.36	***	0.378	***	0.355	***	0.344	***	0.444	***	0.458	***
	IL17A	0.209	***	0.124	***	0.139	*	0.085	0.064	0.049	0.271	0.02	0.657
Treg	FOXP3	0.553	***	0.46	***	0.664	***	0.603	***	0.226	***	0.188	***
	CCR8	0.564	***	0.512	***	0.667	***	0.613	***	0.334	***	0.308	***
	STAT5B	0.349	***	0.335	***	0.371	***	0.407	***	0.434	***	0.434	***
	TGFβ (TGFB1)	0.446	***	0.305	***	0.182	***	0.098	0.032	0.318	***	0.289	***
T‐cell exhaustion	PD‐1 (PDCD1)	0.482	***	0.325	***	0.651	***	0.592	***	0.126	*	0.06	0.186
	CTLA‐4	0.529	***	0.409	***	0.626	***	0.557	***	0.21	***	0.156	**
	LAG3	0.325	***	0.227	***	0.503	***	0.448	***	0.039	0.382	−0.015	0.744
	TIM‐3 (HAVCR2)	0.624	***	0.554	***	0.709	***	0.657	***	0.276	***	0.227	***
	GZMB	0.47	***	0.331	***	0.463	***	0.383	***	−0.03	0.491	−0.101	0.025

Abbreviations: BRCA, breast invasive carcinoma; Cor, R value of Spearman's correlation; LUAD, lung adenocarcinoma; LUSC, lung squamous cell carcinoma; None, correlation without adjustment; Purity, correlation adjusted by purity; TAM, tumour‐associated macrophage; Tfh, follicular helper T cell; Th, T‐helper cell; Treg, regulatory T cell.

*p < 0.01; **p < 0.001; *** *p* < 0.0001.

We further assessed the relationships between *CELF2* and these markers in BRCA, LUSC and LUAD using GEPIA, revealing similar correlations between *CELF2* and T cell (general), monocyte, TAM, M2 macrophage and DC markers to those TIMER (Table 3). Previous studies have demonstrated that the proportion of TAMs in the TME and their polarization status have important effects on cancer growth, invasion, metastasis and drug resistance.^44^, ^45^ Our findings suggested that *CELF2* might modulate BRCA and LUSC progression by affecting macrophage polarization. In addition, the above immune infiltration analysis revealed that elevated *CELF2* expression had a strong correlation with increased CD8+ T cells, DCs and neutrophil infiltration in BRCA, and DC infiltration in LUSC. Consistently, the TIMER and GEPIA results also validated that DC markers such as *HLA*‐*DRA*, *HLA*‐*DPA1*, *NRP1* and *ITGAX* were significantly correlated with *CELF2*. These results further indicated a strong relationship between *CELF2* expression and DCs infiltration. Notably, the marker levels of Th1 (*TBX21* and *STAT4*) and Treg (*FOXP3* and *CCR8*) cells, which are primarily immunosuppressive, were also significantly associated with *CELF2* expression. It has been shown that DCs promote tumour metastasis by decreasing CD8+ T‐cell cytotoxicity and enhancing Treg responses.^4^, ^5^ Recent studies have also showed that an autologous dendritic cell vaccine can kill breast cancer cells by polarizing the Th1 response, which raised new hopes for the treatment and prevention of breast cancer.^46^ However, whether *CELF2* mediates the progression and metastasis of BRCA and LUSC via affecting DC infiltration remains to be further investigated.

**TABLE 3:** Correlation analysis between *CELF2* and relate markers of T cell (general), monocyte, TAM, M2 macrophage and DC in GEPIA

Description	Gene markers	BRCA				LUSC				LUAD			
		Tumour		Normal		Tumour		Normal		Tumour		Normal	
		Cor	*p*	Cor	*p*	Cor	*p*	Cor	*p*	Cor	*p*	Cor	*p*
T cell (general)	CD3D	0.53	***	−0.48	***	0.56	***	−0.33	0.019	0.1	0.021	−0.16	0.23
	CD3E	0.6	***	−0.45	***	0.66	***	−0.1	0.48	0.27	***	0.19	0.15
	CD2	0.6	***	−0.38	***	0.66	***	−0.052	0.72	0.25	***	0.045	0.74
Monocyte	CD86	0.66	***	0.59	***	0.67	***	0.18	0.2	0.29	***	−0.07	0.6
	CD115 (CSF1R)	0.66	***	0.49	***	0.74	***	0.27	0.063	0.37	***	0.16	0.24
TAM	CCL2	0.48	***	0.34	**	0.5	***	−0.0066	0.96	0.15	*	0.14	0.29
	CD68	0.6	***	0.59	***	0.53	***	0.18	0.2	0.31	***	0.039	0.77
	IL10	0.59	***	0.62	***	0.52	***	−0.14	0.33	0.24	***	0.058	0.66
M2 macrophage	CD163	0.52	***	0.61	***	0.67	***	0.25	0.085	0.27	***	0.03	0.82
	VSIG4	0.5	***	0.57	***	0.64	***	0.13	0.37	0.26	***	−0.15	0.27
	MS4A4A	0.64	***	0.67	***	0.65	***	0.049	0.74	0.33	***	−0.17	0.21
Dendritic cell	HLA‐DPB1	0.58	***	−0.29	*	0.77	***	−0.17	0.24	0.37	***	−0.048	0.72
	HLA‐DQB1	0.34	***	−0.091	0.34	0.41	***	−0.13	0.36	0.23	***	0.084	0.52
	HLA‐DRA	0.65	***	0.044	0.64	0.71	***	−0.16	0.27	0.33	***	−0.27	0.038
	HLA‐DPA1	0.64	***	0.028	0.77	0.75	***	0.25	0.078	0.39	***	0.21	0.1
	BDCA‐1 (CD1C)	0.55	***	0.069	0.47	0.49	***	−0.032	0.83	0.37	***	−0.2	0.13
	BDCA‐4 (NRP1)	0.62	***	0.85	***	0.5	***	0.53	***	0.34	***	0.47	**
	CD11C (ITGAX)	0.61	***	0.11	0.26	0.63	***	0.2	0.17	0.27	***	0.13	0.34

Abbreviations: BRCA, breast invasive carcinoma; LUAD, lung adenocarcinoma; LUSC, lung squamous cell carcinoma; Normal, correlation analysis in normal tissue of TCGA; TAM, tumour‐associated macrophage; Tumour, correlation analysis in tumour tissue of TCGA.

*p < 0.01; **p < 0.001; ***p < 0.0001.

### 3.6 Correlations between *CELF2* expression and the responses to immunotherapy

**FIGURE 5:**
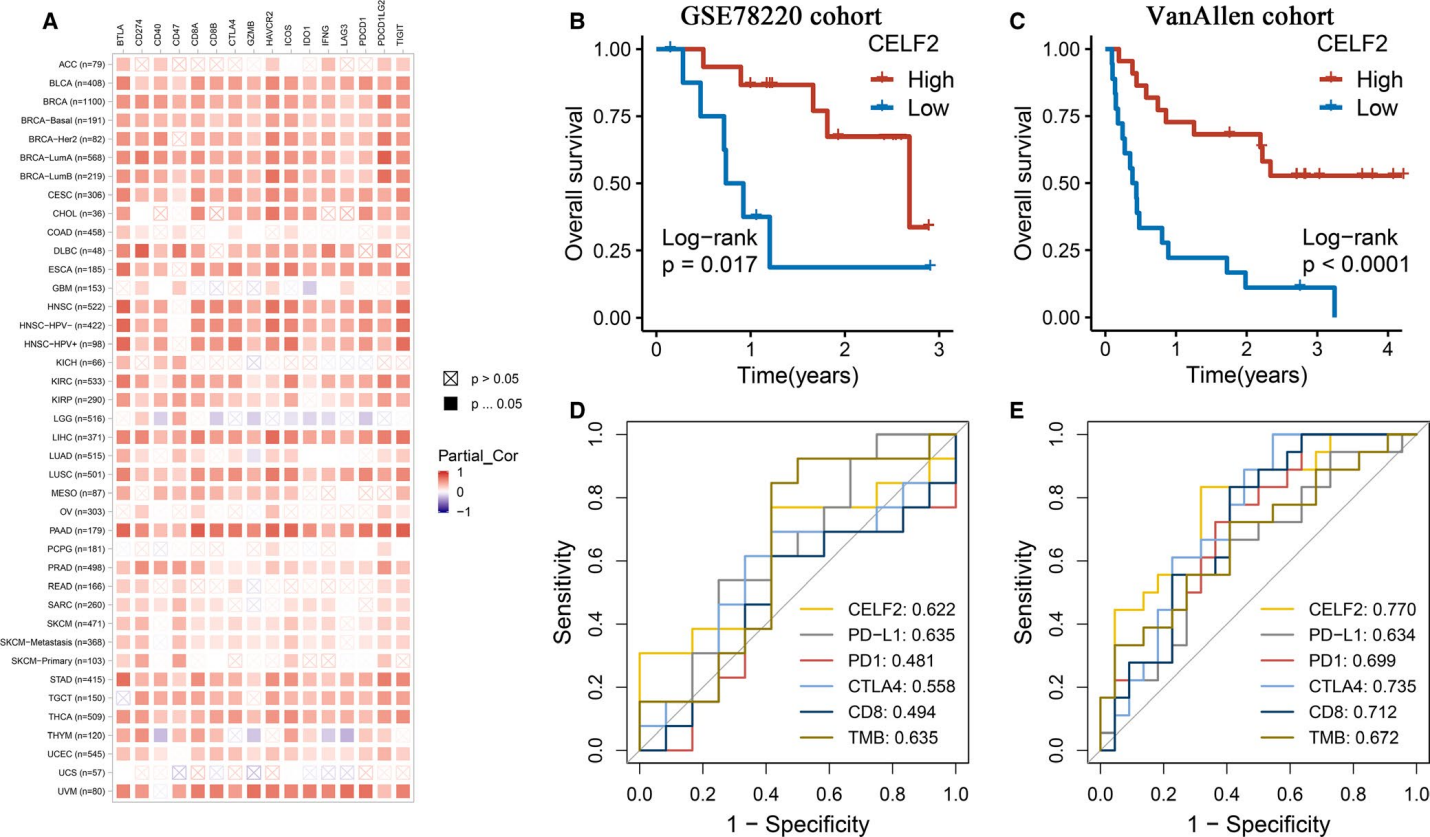
Correlations between *CELF2* and immune checkpoint molecules (ICMs) as well as the responses to immunotherapy in a variety of tumours. (A) Spearman correlations between *CELF2* expression and the expression of ICMs in different cancer types after adjusting for tumour purity. (B, C) Kaplan‐Meier survival analysis of high *CELF2* and low *CELF2* group in GSE78220 cohort (B) and Van Allen cohort (C). (D, E) The ROC curves and AUC values of *CELF2* and five other biomarkers for predicting immunotherapeutic response in GSE78220 cohort (D) and Van Allen cohort (E)

The above showed that *CELF2* expression was significantly associated with marker gene sets of T‐cell exhaustion, such as *PD*‐*1*, *CTLA*‐*4* and *TIM*‐*3*, implying that *CELF2* might play crucial roles in immune tolerance and immune evasion (Table 2). We further explored the relationships between *CELF2* and ICMs, including *BTLA*, *CD274*, *CD40*, *CD47*, *CD8A*, *CD8B*, *CTLA*‐*4*, *GZMB*, *TIM*‐*3* (*HAVCR2*), ICOS, *IDO1*, *IFNG*, *LAG3*, *PDCD1*, *PDCD1LG2* and *TIGIT*.^12^, ^35^, ^36^ The results revealed that *CELF2* expression was significantly positively correlated with the expression of these molecules in BRCA and LUSC (Figure 5A). In addition, we also observed the significant correlations between *CELF2* and ICM expression in urinary system tumours such as BLCA, KIRC and kidney renal papillary cell carcinoma (KIRP), as well as digestive system tumours such as esophageal carcinoma (ESCA), liver hepatocellular carcinoma (LIHC), pancreatic adenocarcinoma (PAAD) and STAD. These results suggested the possibility of *CELF2* as a potential biomarker for ICI‐directed immunotherapies.

To further investigate whether *CELF2* could accurately predict the efficacy to immunotherapy, we enrolled two immunotherapeutic cohorts receiving anti‐PD‐1 therapy. Interestingly, patients with high *CELF2* showed significantly prolonged OS in the two cohorts (Figure 5B,C). We then included the other five widely used immunotherapeutic biomarkers, including *PD*‐*1*, *PD*‐*L1*, *CTLA*‐*4*, *CD8* and TMB. In the GSE78220 cohort of 38 patients, *CELF2* (AUC = 0.622) showed similar predictive power as *PD*‐*L1* (AUC = 0.635) and TMB (AUC = 0.635), better than *CTLA*‐*4*, *CD8* and *PD*‐*1* (AUC = 0.558, 0.494 and 0.481, respectively; Figure 5D). In addition, in another larger Van Allen cohort of 144 patients, the AUC of *CELF2* reached 0.770, which was higher than that of *CTLA*‐*4*, *CD8*, *PD*‐*1*, TMB and *PD*‐*L1* (AUC = 0.735, 0.712, 0.699, 0.672 and 0.634, respectively; Figure 5E). Overall, our study strongly confirmed that *CELF2* could be used to evaluate prognosis and responses to immunotherapy in cancer patients and is superior to remaining prevalent biomarkers.

### 3.7 Experimental verification of *CELF2* expression and prognosis in TNBC and LUSC tissue microarrays

**FIGURE 6:**
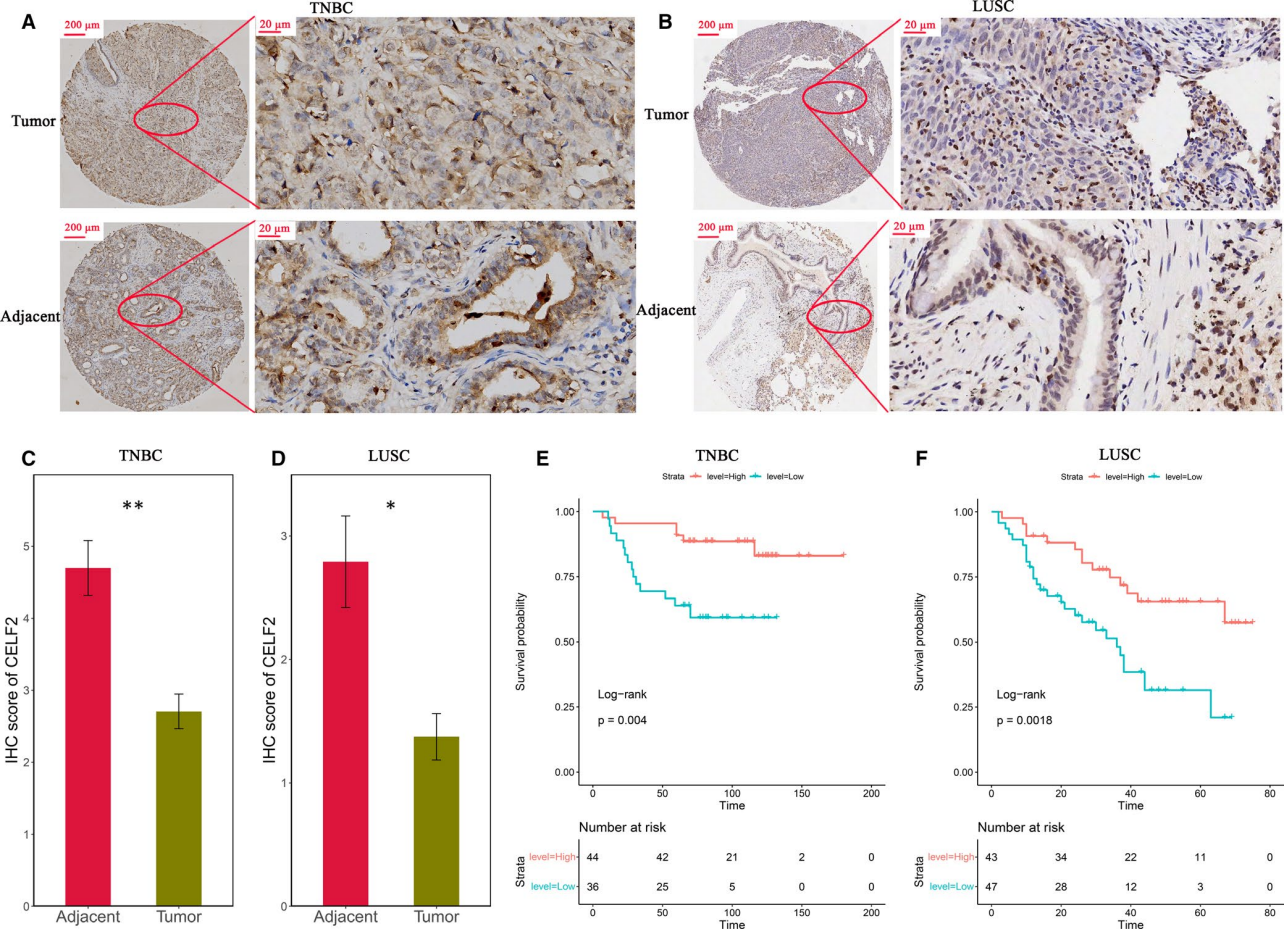
*CELF2* was down‐regulated in triple‐negative breast cancer (TNBC) and lung squamous cell carcinoma (LUSC), as well as predicted better prognosis. (A, B) Representative immunohistochemistry (IHC) staining images of TNBC (A) and LUSC (B) tissue microarrays in tumour tissues and paired adjacent tissues (scale bar: 200 and 20 μm). (C, D) Analysis of IHC scores in TNBC (C) and LUSC (D) tissue microarrays according to *CELF2* staining results. (E, F) Survival rates of tumour patients with high and low protein levels of *CELF2* in TNBC (E) and LUSC (F) tissue microarrays. Kaplan‐Meier method was used to analyse the overall survival (*p < 0.05, **p < 0.01).

The above analysis suggested that *CELF2* has significant prognostic significance in TNBC and LUSC, and is closely related to the TME and immunotherapy efficacy. We further used TNBC (*n* = 80) and LUSC (*n* = 90) tissue microarrays combined with immunohistochemistry for experimental validation at the protein level. The results showed that the protein expression levels of *CELF2* in TNBC and LUSC were significantly reduced compared with the normal tissues (Figure 6A–D). In the TNBC tissue microarray, 44 patients (55%) were classified as the high *CELF2* expression group and 36 patients (45%) as the low *CELF2* expression group according to the optimal cut‐off point of the pathological score, and survival analysis revealed that TNBC patients with high *CELF2* expression had longer OS (*p* = 0.004; Figure 6E). Similarly, the results of LUSC tissue microarray also indicated that the OS of LUSC patients with high *CELF2* expression was significantly prolonged (*p* = 0.0018; Figure 6F). Collectively, these two independent cohorts highlight the significant prognostic significance of *CELF2* in TNBC and LUSC, and the potential possibility of being a candidate biomarker.

## 4 DISCUSSION

In this study, we systematically summarized the expression levels and prognostic value of *CELF2* in diverse cancer types using Oncomine and TIMER database, revealing prominent differences between tumours and adjacent normal tissues. Oncomine analysis showed elevated *CELF2* expression in colorectal, gastric, kidney, leukaemia, liver and melanoma cancers compared with matched normal tissues, whereas *CELF2* expression was down‐regulated in bladder, brain and central nervous system, breast, head and neck, lung, lymphoma, ovarian, prostate and sarcoma cancers (Figure 1A). Based on the RNA‐seq data from the TCGA database, we found that *CELF2* expression was significantly down‐regulated in BLCA, BRCA, COAD, HNSC, LUAD, LUSC, PRAD, READ, STAD, THCA and UCEC relative to their respective adjacent normal tissues, whereas significantly up‐regulated of *CELF2* expression was only found in CHOL, KICH and KIRC (Figure 1B). The inconsistent results of the same cancer in different data sets may be due to the different approaches of data collection in different studies, or the fact that tumours at the same anatomical site contains various histological types in Oncomine database. However, in PrognoScan and Kaplan‐Meier plotter database, we consistently observed that increased *CELF2* expression was associated with better prognosis in breast and lung cancer. Analysis using Kaplan‐Meier plotter indicated that elevated *CELF2* expression correlated with significantly prolonged survival in both breast and lung cancer (Figure 2M–P). Similarly, there were 24 and 15 cohorts in PrognoScan database, respectively, that showed *CELF2* could serve as a predictor of favourable prognosis in breast and lung cancers (Supplementary Table 2). This was consistent with our immunohistochemistry results (Figure 6). Furthermore, high *CELF2* expression was associated with significantly prolonged OS and RFS of breast cancer patients with ER, PR and HER2 negative, lymph node metastasis and higher grade. Notably, *CELF2* expression was much higher in basal‐like breast cancer (also known as TNBC) than in luminal and HER2 subtype (Figure 1B). Briefly, these findings supported that *CELF2* was a potential prognostic biomarker in breast and lung cancers, and might influence TNBC development and metastasis.

Another key finding of this study was that *CELF2* expression correlated with diverse levels of immune infiltration in multiple cancer types, and especially in BRCA and LUSC. Our results demonstrated strong positive relationships between *CELF2* expression and the infiltration levels of DCs, CD8+ T cells and neutrophils in BRCA; meanwhile, *CELF2* in LUSC was strongly positively correlated with DCs (Figure 3A,C). However, we found that *CELF2* expression was weakly correlated with the levels of B cells, CD8+ T cells, CD4+ T cells, macrophages, neutrophils and DCs in LUAD (Figure 3B). These results indicated that *CELF2* expression and the levels of immune infiltration were closely correlated in BRCA and LUSC, but not in LUAD. In addition, we simultaneously observed significant correlation between *CELF2* and certain immunological markers using TIMER and GEPIA database, hinting that *CELF2* could regulate TIIC infiltration and interaction within the TME in BRCA and LUSC (Tables 2 and 3). For example, the markers of monocytes (*CD86* and *CSF1R*) and M2 macrophages (*CD163* and *MS4A4A*) showed strong correlations with *CELF2*, while TAM markers (*CCL2*, *CD68* and *IL10*) showed moderate correlations (Tables 2 and 3). These results revealed a potential role of *CELF2* in modulating TAM polarization. Remarkably, immune infiltration and immune marker set analysis consistently showed a strong correlation between *CELF2* and DCs in BRCA and LUSC (Figure 3A,C, Tables 2 and 3). Combined with the indispensable role of DCs in anti‐tumour immunity and the promising future of DC vaccines in tumour treatment, we are confident that clarifying the mechanism by which *CELF2* interacts with DCs in the TME may provide a new target for immunotherapy.^46^, ^47^, ^48^

Moreover, we further found that *CELF2* in BRCA and LUSC were moderately to strongly correlated with the main immunosuppressive Th1 and Treg cell marker sets (*TBX21* and *STAT4*; *FOXP3* and *CCR8*) as well as T‐cell exhaustion markers (*PD*‐*1*, *CTLA*‐*4* and *TIM*‐*3*) within the TME (Tables 2 and 3). These results might indicate that *CELF2* could regulate T cell‐mediated immunity via Treg and Th1 cell in BRCA and LUSC. Given the lack of reliable diagnostic and prognostic biomarkers as well as therapeutic targets, TNBC and LUSC treatment remains challenging, whereas immunotherapy offers patients new hope.^7^, ^8^ A recent phase III clinical study on advanced TNBC showed that the levels of stromal tumour‐infiltrating lymphocytes (sTILs) were correlated with PD‐L1 status, and obvious improvements in the efficacy of immunotherapy were observed only in CD8+ and sTILs+ patients who were also PD‐L1+.^40^ Of particular interest was the significant positive correlations between *CELF2* and ICMs in BRCA, LUSC as well as digestive and urinary tumours (Figure 5A). Further exploration also demonstrated that *CELF2* was more accurate than five prevalent indicators including *PD*‐*1*, *PD*‐*L1*, *CTLA*‐*4*, *CD8* and TMB in predicting the responses to immunotherapy, hinting that *CELF2* was a promising biomarker for selecting immunotherapy‐sensitive patients (Figure 5D,E).

To the best of our knowledge, our study is the first to systematically address the expression and prognostic landscape of *CELF2*, which plays an indispensable in RNA processing, and to explore its potential relationship with immune infiltration in pan‐cancer. Second, we comprehensively analysed a large amount of data from the multiple public databases and our two tissue microarrays and validated the results by integrating immune infiltration analysis and correlation analysis of immune marker sets to increase the reliability of our conclusions. Most importantly, we confirmed that *CELF2* could effectively predict the prognosis and responses to immunotherapy in TNBC and LUSC patients, and had significant clinical translational value for TNBC and LUSC that owing poor prognosis due to lack of effective biomarkers and targets. Nevertheless, this study also had several limitations. For example, although we found that *CELF2* expression was associated with the abundance of TIIC infiltration in BRCA and LUSC patients, we could not conclude whether *CELF2* directly affected patient survival through immune infiltration. To overcome these limitations, future detailed molecular and cellular mechanistic studies of *CELF2* and prospective studies including *CELF2* expression, immune cells infiltration, and efficacy of immunotherapy in tumour patients will help provide clear answers.

In summary, elevated *CELF2* expression is correlated with better prognosis and higher TIIC infiltration in a variety of tumours. Especially, for BRCA and LUSC, *CELF2* may contribute to TAM polarization, participate in the interaction between DCs and TME, and regulate immune tolerance and immune escape through Treg and Th1 cells. Furthermore, we demonstrated that *CELF2* is strongly correlated with ICMs in various tumours, and significantly outperforms five prevalent biomarkers in predicting the responses to immunotherapy. Therefore, *CELF2* may be a crucial regulator of tumour immune cell infiltration and serve as a prognostic and immunotherapeutic biomarker in TNBC and LUSC.

## CONFLICT OF INTEREST

The authors declare that they have no conflict of interest.

## AUTHOR CONTRIBUTIONS

YLS, XWH, JZ and LFL: Research design. LBW and ZQL: Data mining and data analysis. LL, CGG and DCJ: Assistance with data mining and data analysis. LBW, ZQL and LFL: Writing the manuscript. LBW, ZQL, XWH and YLS: Editing and revision of the manuscript. All authors approved the final version of the manuscript.

## ETHICAL APPROVAL

The procedures used in the present study were approved (approval no. YB M‑05‑02 V.2) by the Ethics Committee of the Shanghai Outdo Biotech Company, a member of the National Human Genetic Resources Sharing Service Platform (Shanghai, China) and were performed in accordance with the ethical standards of the Institutional and National Research Committee and with the Declaration of Helsinki. Written informed consent was acquired from all patients.

## Supporting information

Supplementary Material

## Data Availability

All data generated or analysed during this study are included in this article.
