# Supplementary material for: CELF2 is a candidate prognostic and immunotherapy biomarker in triple‐negative breast cancer and lung squamous cell carcinoma: A pan‐cancer analysis
Source: J Cell Mol Med. 2021 Jul 19;25(15):7559–74. doi: 10.1111/jcmm.16791 (PMC8335674; doi:10.1111/jcmm.16791)
Supplement: Supplementary file 1 — Supplementary Material [file JCMM-25-7559-s001.docx]

**Supplementary Materials**

***CELF2* is a candidate prognostic and immunotherapy biomarker in triple-negative breast cancer and lung squamous cell carcinoma: A pan-cancer analysis**

**Supplementary Table 1. CELF2 expression in cancers versus normal tissue in Oncomine database**

| Cancer | Cancer type | P-value | Fold change | Rank (%) | Sample | Reference (PMID) |
| --- | --- | --- | --- | --- | --- | --- |
| Bladder | Infiltrating Bladder Urothelial Carcinoma | 7.95E-23 | -2.941 | 1% | 129 | 16432078 |
|  | Superficial Bladder Cancer | 3.34E-18 | -5.729 | 2% | 76 | 16432078 |
|  | Superficial Bladder Cancer | 2.89E-16 | -2.629 | 1% | 194 | 20421545 |
|  | Infiltrating Bladder Urothelial Carcinoma | 1.25E-09 | -2.111 | 2% | 150 | 20421545 |
| Brain and CNS | Glioblastoma | 3.01E-11 | -2.33 | 2% | 25 | 16697959 |
|  | Glioblastoma | 2.82E-09 | -3.704 | 3% | 31 | 16204036 |
|  | Diffuse Astrocytoma | 4.15E-04 | -2.017 | 3% | 30 | 16616334 |
|  | Anaplastic Astrocytoma | 1.84E-7 | -2.08 | 6% | 42 | 16616334 |
|  | Glioblastoma | 1.96E-12 | -2.696 | 11% | 104 | 16616334 |
|  | Oligodendroglioma | 4.76E-08 | -1.912 | 12% | 73 | 16616334 |
|  | Glioblastoma | 3.88E-05 | -3.025 | 5% | 84 | 18565887 |
|  | Brain Glioblastoma | 6.84E-09 | -2.181 | 8% | 552 | TCGA |
| Breast | Invasive Breast Carcinoma Stroma | 9.70E-15 | 6.857 | 6% | 59 | 18438415 |
|  | Tubular Breast Carcinoma | 8.02E-45 | -2.318 | 1% | 211 | 22522925 |
|  | Invasive Ductal and Invasive Lobular Breast Carcinoma | 1.45E-37 | -2.172 | 2% | 234 | 22522925 |
|  | Invasive Breast Carcinoma | 2.32E-10 | -2.219 | 3% | 165 | 22522925 |
|  | Mucinous Breast Carcinoma | 1.27E-21 | -2.362 | 3% | 190 | 22522925 |
|  | Invasive Lobular Breast Carcinoma | 1.20E-36 | -2.007 | 4% | 292 | 22522925 |
|  | Invasive Ductal Breast Carcinoma | 1.15E-50 | -2.333 | 5% | 1700 | 22522925 |
|  | Breast Carcinoma | 1.31E-05 | -2.081 | 7% | 158 | 22522925 |
|  | Ductal Breast Carcinoma in Situ | 2.61E-04 | -1.924 | 9% | 154 | 22522925 |
|  | Medullary Breast Carcinoma | 6.95E-09 | -1.727 | 9% | 176 | 22522925 |
|  | Mixed Lobular and Ductal Breast Carcinoma | 1.59E-06 | -3.164 | 3% | 68 | TCGA |
|  | Mucinous Breast Carcinoma | 3.26E-05 | -4.657 | 3% | 65 | TCGA |
|  | Invasive Ductal Breast Carcinoma | 3.01E-32 | -4.434 | 3% | 450 | TCGA |
|  | Invasive Lobular Breast Carcinoma | 2.01E-12 | -2.622 | 4% | 97 | TCGA |
|  | Invasive Breast Carcinoma | 1.11E-15 | -2.779 | 6% | 137 | TCGA |
|  | Ductal Breast Carcinoma | 8.38E-04 | -2.158 | 13% | 47 | 16473279 |
| Colorectal | Colorectal Adenoma Epithelia | 2.81E-04 | 1.608 | 5% | 78 | 18403596 |
|  | Colon Adenoma | 4.98E-07 | 2.993 | 3% | 15 | 20957034 |
|  | Colon Adenoma Epithelia | 1.71E-05 | 2.233 | 6% | 15 | 20957034 |
|  | Colon Carcinoma | 9.38E-07 | 1.662 | 9% | 15 | 20957034 |
|  | Colon Carcinoma Epithelia | 4.36E-05 | 1.623 | 12% | 15 | 20957034 |
|  | Colon Adenoma | 9.45E-11 | 2.086 | 7% | 57 | 18171984 |
|  | Colorectal Adenocarcinoma | 7.12E-07 | 1.611 | 9% | 69 | 20957034 |
|  | Colorectal Carcinoma | 2.18E-05 | 1.536 | 18% | 82 | 20143136 |
| Gastric | Diffuse Gastric Adenocarcinoma | 4.14E-04 | 1.589 | 3% | 37 | 19081245 |
| Head and Neck | Tongue Squamous Cell Carcinoma | 1.46E-04 | -2.77 | 2% | 38 | 18254958 |
|  | Tongue Squamous Cell Carcinoma | 4.64E-04 | -1.662 | 11% | 57 | 19138406 |
| Kidney | Clear Cell Renal Cell Carcinoma | 7.59E-11 | 3.558 | 1% | 31 | 19445733 |
|  | Renal Oncocytoma | 4.69E-06 | 7.605 | 1% | 9 | 19445733 |
|  | Papillary Renal Cell Carcinoma | 2.77E-05 | 2.111 | 2% | 24 | 19445733 |
|  | Clear Cell Sarcoma of the Kidney | 9.35E-07 | 4.496 | 1% | 17 | 16299227 |
|  | Clear Cell Renal Cell Carcinoma | 1.08E-06 | 2.571 | 3% | 20 | 17699851 |
|  | Papillary Renal Cell Carcinoma | 6.76E-11 | 2.721 | 4% | 34 | 16115910 |
|  | Clear Cell Renal Cell Carcinoma | 2.77E-11 | 2.67 | 6% | 46 | 16115910 |
|  | Renal Oncocytoma | 8.85E-07 | 4.07 | 9% | 35 | 16115910 |
|  | Non-Hereditary Clear Cell Renal Cell Carcinoma | 4.98E-07 | 2.63 | 6% | 38 | 19470766 |
|  | Hereditary Clear Cell Renal Cell Carcinoma | 6.75E-08 | 3.061 | 7% | 43 | 19470766 |
|  | Clear Cell Renal Cell Carcinoma | 8.56E-04 | 2.502 | 5% | 18 | 14641932 |
|  | Renal Wilms Tumor | 1.08E-04 | -2.982 | 3% | 21 | 16299227 |
| Leukemia | Acute Myeloid Leukemia | 2.04E-09 | 3.504 | 2% | 27 | 17410184 |
|  | Chronic Lymphocytic Leukemia | 2.49E-04 | 2.321 | 10% | 59 | 15778709 |
|  | B-Cell Childhood Acute Lymphoblastic Leukemia | 2.18E-16 | 1.586 | 13% | 633 | 20406941 |
|  | B-Cell Acute Lymphoblastic Leukemia | 1.95E-12 | 1.507 | 15% | 221 | 20406941 |
|  | T-Cell Acute Lymphoblastic Leukemia | 4.27E-13 | 1.521 | 15% | 248 | 20406941 |
|  | Acute Myeloid Leukemia | 8.44E-07 | -1.54 | 6% | 29 | 17410184 |
|  | B-Cell Acute Lymphoblastic Leukemia | 3.68E-08 | -1.572 | 8% | 93 | 17410184 |
|  | T-Cell Acute Lymphoblastic Leukemia | 2.44E-04 | -2.198 | 17% | 17 | 17410184 |
| Liver | Cirrhosis | 8.51E-27 | 2.618 | 1% | 77 | 19098997 |
|  | Hepatocellular Carcinoma | 5.81E-09 | 2.532 | 4% | 57 | 19098997 |
|  | Cirrhosis | 1.76E-07 | 2.084 | 1% | 23 | 17393520 |
| Lung | Squamous Cell Lung Carcinoma | 5.81E-07 | -2.189 | 1% | 10 | 16188928 |
|  | Lung Adenocarcinoma | 9.90E-28 | -1.744 | 1% | 246 | 22080568 |
|  | Lung Adenocarcinoma | 1.80E-41 | -3.649 | 1% | 116 | 22613842 |
|  | Lung Adenocarcinoma | 7.17E-14 | -2.487 | 1% | 96 | 12118244 |
|  | Squamous Cell Lung Carcinoma | 6.72E-07 | -3.893 | 2% | 19 | 11707590 |
|  | Lung Adenocarcinoma | 1.25E-05 | -1.98 | 3% | 45 | 11707590 |
|  | Lung Adenocarcinoma | 2.19E-18 | -3.511 | 1% | 110 | 20421987 |
|  | Squamous Cell Lung Carcinoma | 3.22E-20 | -6.031 | 2% | 92 | 20421987 |
|  | Large Cell Lung Carcinoma | 2.55E-13 | -2.059 | 2% | 84 | 20421987 |
|  | Squamous Cell Lung Carcinoma | 5.81E-06 | -8.976 | 3% | 38 | 11707567 |
|  | Lung Carcinoid Tumor | 9.41E-09 | -21.478 | 4% | 37 | 11707567 |
|  | Lung Adenocarcinoma | 6.33E-04 | -3.632 | 12% | 149 | 11707567 |
|  | Lung Adenocarcinoma | 1.47E-15 | -1.824 | 5% | 107 | 18297132 |
|  | Lung Adenocarcinoma | 1.28E-07 | -2.278 | 5% | 57 | 17540040 |
| Lymphoma | Mantle Cell Lymphoma | 2.70E-04 | 2.317 | 9% | 33 | 15778709 |
|  | Unspecified Peripheral T-Cell Lymphoma | 3.20E-05 | 1.527 | 21% | 48 | 17304354 |
|  | Follicular Lymphoma | 5.57E-04 | 2.064 | 22% | 58 | 19412164 |
|  | Primary Cutaneous Anaplastic Large Cell Lymphoma | 4.51E-10 | -6.984 | 1% | 48 | 19657361 |
|  | Classical Hodgkin's Lymphoma | 1.18E-07 | -4.594 | 1% | 45 | 19657361 |
|  | Anaplastic Large Cell Lymphoma, ALK-Negative | 1.74E-04 | -1.83 | 3% | 45 | 19657361 |
|  | Anaplastic Large Cell Lymphoma, ALK-Positive | 7.31E-04 | -7.683 | 6% | 46 | 19657361 |
|  | Burkitt's Lymphoma | 6.90E-08 | -2.897 | 2% | 30 | 18794340 |
|  | Follicular Lymphoma | 1.46E-04 | -2.155 | 2% | 30 | 18794340 |
|  | Diffuse Large B-Cell Lymphoma | 1.13E-04 | -2.067 | 6% | 36 | 18794340 |
|  | Germinal Center B-Cell-Like Diffuse Large B-Cell Lymphoma | 3.24E-05 | -2.131 | 11% | 29 | 19412164 |
|  | Activated B-Cell-Like Diffuse Large B-Cell Lymphoma | 6.34E-05 | -2.005 | 23% | 37 | 19412164 |
|  | Diffuse Large B-Cell Lymphoma | 3.38E-04 | -1.67 | 36% | 64 | 19412164 |
|  | Follicular Lymphoma | 8.59E-04 | -1.537 | 40% | 58 | 19412164 |
| Melanoma | Cutaneous Melanoma | 5.46E-05 | 1.989 | 1% | 18 | 18442402 |
| Ovarian | Ovarian Clear Cell Adenocarcinoma | 9.02E-05 | -1.604 | 1% | 12 | 15161682 |
|  | Ovarian Endometrioid Adenocarcinoma | 1.35E-04 | -1.554 | 1% | 14 | 15161682 |
|  | Ovarian Serous Cystadenocarcinoma | 1.72E-08 | -3.331 | 1% | 594 | TCGA |
|  | Ovarian Serous Adenocarcinoma | 6.83E-17 | -10.439 | 1% | 53 | 19486012 |
|  | Ovarian Carcinoma | 2.91E-09 | -4.756 | 3% | 195 | 18593951 |
|  | Ovarian Serous Surface Papillary Carcinoma | 4.79E-04 | -5.108 | 11% | 32 | 11158614 |
|  | Ovarian Mucinous Adenocarcinoma | 7.26E-05 | -1.543 | 11% | 17 | 16452189 |
| Prostate | Prostate Carcinoma | 1.13E-06 | -10.743 | 1% | 34 | 11507037 |
|  | Prostate Carcinoma | 3.49E-05 | -3.057 | 1% | 21 | 19737960 |
|  | Prostate Carcinoma | 2.95E-12 | -1.676 | 2% | 101 | 14711987 |
|  | Prostate Carcinoma | 7.48E-04 | -2.209 | 3% | 102 | 12086878 |
|  | Prostate Carcinoma | 2.80E-06 | -1.634 | 6% | 87 | 22722839 |
| Sarcoma | Clear Cell Sarcoma of the Kidney | 9.35E-07 | 4.496 | 1% | 17 | 16299227 |
|  | Leiomyosarcoma | 7.42E-04 | 3.045 | 7% | 21 | 15994966 |
|  | Myxoid/Round Cell Liposarcoma | 3.39E-10 | -2.685 | 2% | 29 | 20601955 |
|  | Dedifferentiated Liposarcoma | 5.24E-09 | -1.884 | 2% | 55 | 20601955 |
|  | Leiomyosarcoma | 2.71E-08 | -1.598 | 2% | 35 | 20601955 |
|  | Myxofibrosarcoma | 1.89E-07 | -1.848 | 3% | 40 | 20601955 |
|  | Pleomorphic Liposarcoma | 3.30E-05 | -1.795 | 6% | 32 | 20601955 |
| Other | Skin Basal Cell Carcinoma | 1.67E-05 | 2.384 | 3% | 19 | 18442402 |
|  | Teratoma, NOS | 4.07E-07 | 2.181 | 6% | 20 | 16424014 |
|  | Embryonal Carcinoma, NOS | 1.30E-05 | 1.54 | 13% | 21 | 16424014 |
|  | Pleural Malignant Mesothelioma | 7.12E-04 | 2.111 | 9% | 49 | 15920167 |

**Supplementary Table 2. Relation between CELF2 expression and patient prognosis of different cancer in PrognoScan database.**

| Cancer type | Dataset | Endpoint | N | Hazard ratio(95%CI) | Cox P |
| --- | --- | --- | --- | --- | --- |
| Blood cancer | GSE12417-GPL570 | Overall Survival | 79 | 1.94 [1.11 - 3.36] | 0.019049 |
|  | GSE5122 | Overall Survival | 58 | 1.56 [1.00 - 2.41] | 0.047528 |
|  | GSE5122 | Overall Survival | 58 | 1.80 [1.06 - 3.06] | 0.02959 |
|  | E-TABM-346 | Overall Survival | 53 | 0.57 [0.34 - 0.97] | 0.03698 |
| Brain cancer | GSE4271-GPL96 | Overall Survival | 77 | 0.36 [0.17 - 0.77] | 0.008263 |
|  | GSE4271-GPL96 | Overall Survival | 77 | 0.44 [0.26 - 0.73] | 0.001652 |
|  | GSE4271-GPL96 | Overall Survival | 77 | 0.41 [0.23 - 0.72] | 0.00216 |
|  | GSE4271-GPL97 | Overall Survival | 77 | 0.39 [0.20 - 0.74] | 0.004009 |
|  | GSE4271-GPL97 | Overall Survival | 77 | 0.48 [0.29 - 0.80] | 0.004608 |
|  | MGH-glioma | Overall Survival | 50 | 0.15 [0.04 - 0.53] | 0.003125 |
|  | GSE4412-GPL96 | Overall Survival | 74 | 0.34 [0.15 - 0.77] | 0.010075 |
| Breast cancer | GSE19615 | Distant Metastasis Free Survival | 115 | 0.28 [0.12 - 0.67] | 0.004072 |
|  | GSE19615 | Distant Metastasis Free Survival | 115 | 0.28 [0.10 - 0.78] | 0.015044 |
|  | GSE19615 | Distant Metastasis Free Survival | 115 | 0.16 [0.04 - 0.59] | 0.005671 |
|  | GSE19615 | Distant Metastasis Free Survival | 115 | 0.28 [0.12 - 0.66] | 0.003723 |
|  | GSE6532-GPL570 | Distant Metastasis Free Survival | 87 | 0.53 [0.30 - 0.94] | 0.02856 |
|  | GSE6532-GPL570 | Relapse Free Survival | 87 | 0.64 [0.42 - 0.99] | 0.046217 |
|  | GSE6532-GPL570 | Relapse Free Survival | 87 | 0.53 [0.30 - 0.94] | 0.02856 |
|  | GSE6532-GPL570 | Distant Metastasis Free Survival | 87 | 0.64 [0.42 - 0.99] | 0.046217 |
|  | GSE9195 | Relapse Free Survival | 77 | 12.15 [1.04 - 141.35] | 0.046045 |
|  | GSE9195 | Relapse Free Survival | 77 | 4.19 [1.47 - 11.96] | 0.007517 |
|  | GSE9195 | Distant Metastasis Free Survival | 77 | 4.19 [1.25 - 14.10] | 0.020494 |
|  | GSE11121 | Distant Metastasis Free Survival | 200 | 0.53 [0.28 - 0.99] | 0.047174 |
|  | GSE9893 | Overall Survival | 155 | 1.78 [1.07 - 2.95] | 0.025525 |
|  | GSE1456-GPL96 | Disease Specific Survival | 159 | 0.46 [0.25 - 0.81] | 0.008005 |
|  | GSE1456-GPL96 | Disease Specific Survival | 159 | 0.49 [0.25 - 0.96] | 0.036896 |
|  | GSE1456-GPL96 | Overall Survival | 159 | 0.50 [0.30 - 0.82] | 0.005745 |
|  | GSE1456-GPL96 | Overall Survival | 159 | 0.50 [0.28 - 0.90] | 0.01971 |
|  | GSE1456-GPL96 | Relapse Free Survival | 159 | 0.55 [0.33 - 0.90] | 0.017097 |
|  | GSE1456-GPL97 | Relapse Free Survival | 159 | 0.36 [0.14 - 0.94] | 0.037642 |
|  | GSE1456-GPL97 | Disease Specific Survival | 159 | 0.29 [0.09 - 0.91] | 0.033495 |
|  | GSE3494-GPL96 | Disease Specific Survival | 236 | 0.43 [0.25 - 0.74] | 0.002519 |
|  | GSE3494-GPL96 | Disease Specific Survival | 236 | 0.54 [0.33 - 0.88] | 0.013186 |
|  | GSE3494-GPL96 | Disease Specific Survival | 236 | 0.54 [0.38 - 0.75] | 0.000332 |
|  | GSE3494-GPL97 | Disease Specific Survival | 236 | 0.49 [0.25 - 0.96] | 0.037489 |
|  | GSE4922-GPL96 | Disease Free Survival | 249 | 0.64 [0.43 - 0.96] | 0.031589 |
|  | GSE4922-GPL96 | Disease Free Survival | 249 | 0.67 [0.49 - 0.90] | 0.008119 |
|  | GSE4922-GPL96 | Disease Free Survival | 249 | 0.63 [0.40 - 0.99] | 0.043655 |
|  | GSE7390 | Distant Metastasis Free Survival | 198 | 0.78 [0.61 - 1.00] | 0.045967 |
| Colorectal cancer | GSE17536 | Disease Specific Survival | 177 | 3.64 [1.47 - 8.98] | 0.005112 |
|  | GSE17536 | Disease Specific Survival | 177 | 1.84 [1.07 - 3.18] | 0.028345 |
|  | GSE17537 | Disease Free Survival | 55 | 0.05 [0.00 - 0.57] | 0.016188 |
|  | GSE17537 | Disease Specific Survival | 49 | 0.03 [0.00 - 0.83] | 0.038616 |
| Eye cancer | GSE22138 | Distant Metastasis Free Survival | 63 | 1.33 [1.10 - 1.60] | 0.002774 |
|  | GSE22138 | Distant Metastasis Free Survival | 63 | 1.34 [1.10 - 1.62] | 0.002929 |
|  | GSE22138 | Distant Metastasis Free Survival | 63 | 1.28 [1.04 - 1.57] | 0.021199 |
|  | GSE22138 | Distant Metastasis Free Survival | 63 | 1.32 [1.11 - 1.57] | 0.001588 |
| Lung cancer | jacob-00182-CANDF | Overall Survival | 82 | 0.56 [0.33 - 0.93] | 0.024096 |
|  | jacob-00182-CANDF | Overall Survival | 82 | 0.58 [0.36 - 0.95] | 0.030227 |
|  | jacob-00182-CANDF | Overall Survival | 82 | 0.48 [0.27 - 0.85] | 0.012268 |
|  | HARVARD-LC | Overall Survival | 84 | 0.56 [0.34 - 0.92] | 0.021587 |
|  | GSE13213 | Overall Survival | 117 | 0.74 [0.57 - 0.96] | 0.020902 |
|  | GSE31210 | Overall Survival | 204 | 0.48 [0.25 - 0.92] | 0.026603 |
|  | GSE31210 | Relapse Free Survival | 204 | 0.41 [0.24 - 0.70] | 0.001043 |
|  | GSE31210 | Relapse Free Survival | 204 | 0.39 [0.24 - 0.63] | 9.53E-05 |
|  | GSE31210 | Overall Survival | 204 | 0.48 [0.28 - 0.80] | 0.005145 |
|  | GSE31210 | Relapse Free Survival | 204 | 0.41 [0.28 - 0.62] | 1.41E-05 |
|  | GSE31210 | Relapse Free Survival | 204 | 0.40 [0.24 - 0.67] | 0.000538 |
|  | GSE31210 | Overall Survival | 204 | 0.52 [0.30 - 0.91] | 0.020813 |
|  | GSE31210 | Relapse Free Survival | 204 | 0.47 [0.31 - 0.71] | 0.000352 |
|  | GSE31210 | Relapse Free Survival | 204 | 0.44 [0.27 - 0.73] | 0.001484 |
|  | GSE4573 | Overall Survival | 129 | 0.63 [0.41 - 0.97] | 0.03536 |
| Ovarian cancer | DUKE-OC | Overall Survival | 133 | 0.83 [0.69 - 0.99] | 0.038686 |
| Skin cancer | GSE19234 | Overall Survival | 38 | 0.32 [0.12 - 0.83] | 0.019353 |
| Soft tissue cancer | GSE30929 | Distant Recurrence Free Survival | 140 | 0.62 [0.47 - 0.83] | 0.001326 |
|  | GSE30929 | Distant Recurrence Free Survival | 140 | 0.52 [0.36 - 0.76] | 0.000658 |
|  | GSE30929 | Distant Recurrence Free Survival | 140 | 0.59 [0.44 - 0.80] | 0.000508 |

**Supplementary Figure 1. Correlation of CELF2 expression with immune infiltration level in diverse type cancers via TIMER database.**


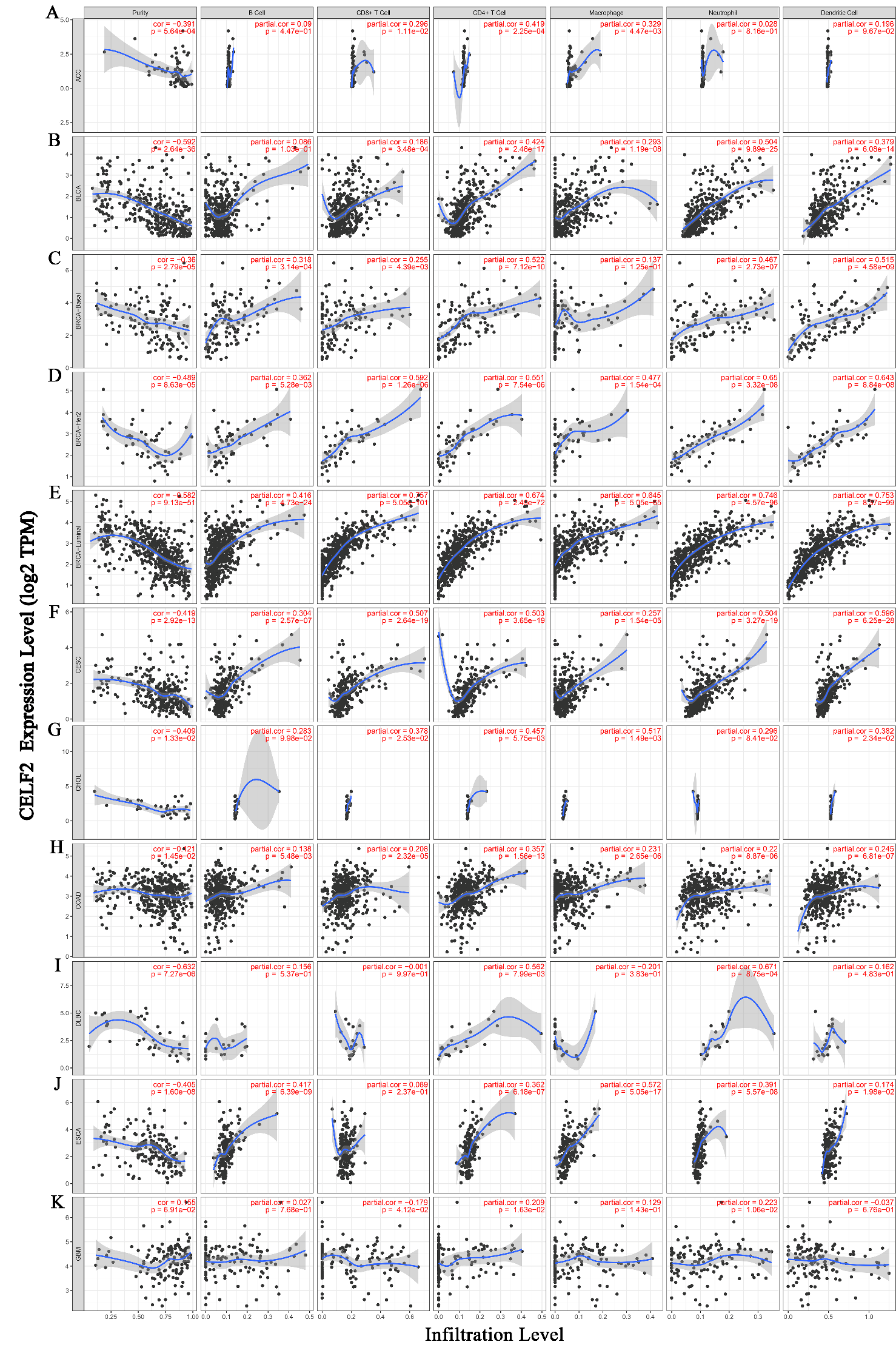


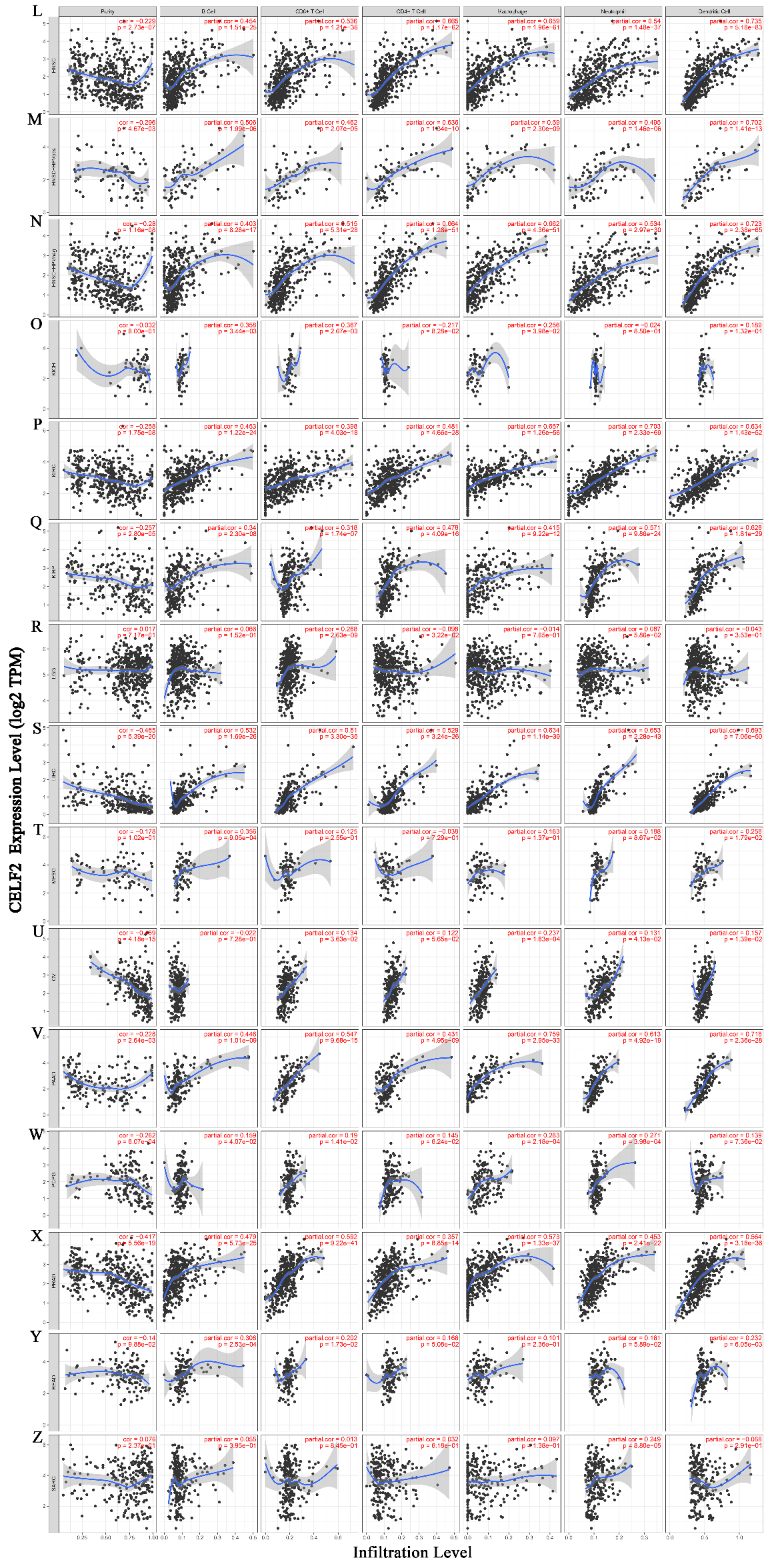


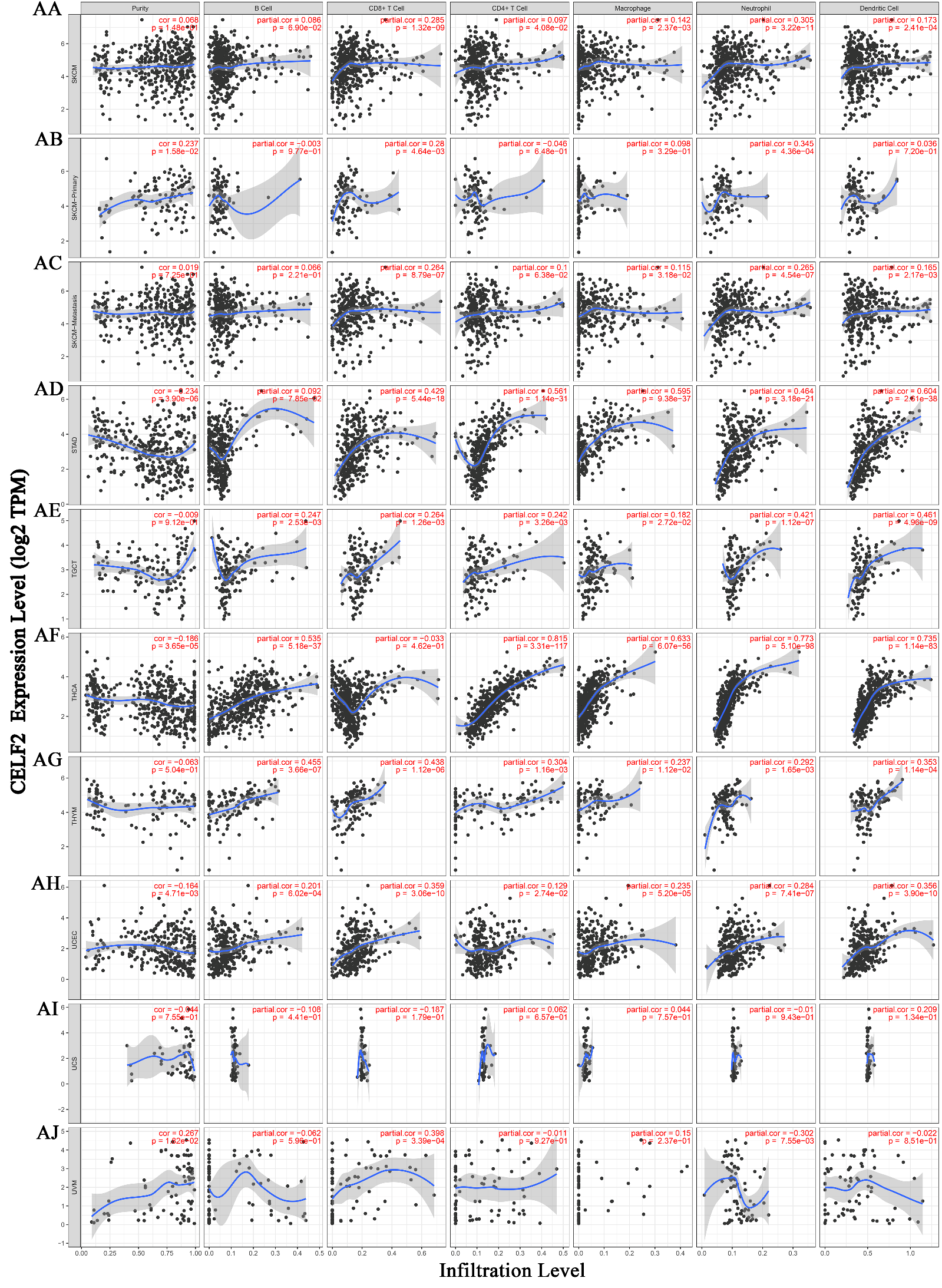


**Supplementary Figure 1.** Correlation of CELF2 expression with immune infiltration levels in Adrenocortical carcinoma (ACC) (**A**), Bladder Urothelial Carcinoma (BLCA) (**B**), Breast invasive carcinoma-Basal (BRCA-Basal) (**C**), Breast invasive carcinoma-Her2 (BRCA-Her2) (**D**),Breast invasive carcinoma-Luminal (BRCA-Luminal) (**E**), Cervical squamous cell carcinoma and endocervical adenocarcinoma (CESC) (**F**), Cholangiocarcinoma (CHOL) (**G**), Colon adenocarcinoma (COAD)(**H**),Lymphoid Neoplasm Diffuse Large B-cell Lymphoma (DLBC) (**I**), Esophageal carcinoma (ESCA) (**J**), Glioblastoma multiforme (GBM) (**K**) ,Head and Neck squamous cell carcinoma (HNSC) (**L**), Head and Neck squamous cell carcinoma-HPVpos(HNSC-HPVpos) (**M**) , Head and Neck squamous cell carcinoma-HPVneg(HNSC-HPVneg) (**N**),Kidney Chromophobe(KICH) (**O**), Kidney renal clear cell carcinoma(KIRC) (**P**), Kidney renal papillary cell carcinoma(KIRP) (**Q**), Brain Lower Grade Glioma(LGG) (**R**), Liver hepatocellular carcinoma(LIHC) (**S**),Mesothelioma(MESO) (**T**), Ovarian serous cystadenocarcinoma (OV) (**U**),Pancreatic adenocarcinoma(PAAD) (**V**), Pheochromocytoma and Paraganglioma (PCPG) (**W**), Prostate adenocarcinoma(PRAD) (**X**), Rectum adenocarcinoma(READ) (**Y**), Sarcoma(SARC) (**Z**), Skin Cutaneous Melanoma(SKCM) (**AA**), Skin Cutaneous Melanoma-Primary (SKCM-Primary) (**AB**), Skin Cutaneous Melanoma-Metastasis(SKCM- Metastasis) (**AC**), Stomach adenocarcinoma (STAD) (**AD**),Testicular Germ Cell Tumors (TGCT) (**AE**), Thyroid carcinoma(THCA) (**AF**), Thymoma(THYM) (**AG**), Uterine Corpus Endometrial Carcinoma(UCEC) (**AH**), Uterine Carcinosarcoma (UCS) (**AI**), Uveal Melanoma(UVM) (**AJ**).
